# Facilitators and barriers of healthcare workers’ recommendation of HPV vaccine for adolescents in Nigeria: views through the lens of theoretical domains framework

**DOI:** 10.1186/s12913-022-08224-7

**Published:** 2022-06-25

**Authors:** Folusho M. Balogun, Olayemi O. Omotade

**Affiliations:** 1 Institute of Child Health, College of Medicine, University of Ibadan, PMB 5116, Oyo 200001 Ibadan, Nigeria; 2 Institute of Child Health, University College Hospital, Ibadan, Nigeria

**Keywords:** Theoretical domain framework, Health care workers, Human Papillomavirus vaccine, Adolescent immunization

## Abstract

**Background:**

The human Papillomavirus (HPV) vaccine has demonstrated efficacy in the prevention of cervical cancer when given in early adolescence. The recommendation of the vaccine by health care workers (HCW) is crucial to the uptake of the vaccine by adolescents and the process of this recommendation is important as it determines subsequent uptake of the vaccine. Understanding of the facilitators and barriers of recommendation of this vaccine can help in the development of strategies to improve its recommendation rates and uptake. This study therefore explored the facilitators and barriers for the recommendation of HPV vaccine for adolescents by HCW in Ibadan, Nigeria using the Theoretical Domain Framework (TDF).

**Methods:**

Key informant interviews were conducted with 14 purposively selected HCW who were in charge of vaccination. Content analysis was used after deductive coding of the data using the domains of the TDF. Relevant concepts for facilitators and barriers of HPV vaccine recommendation and quotes were then identified.

**Results:**

Mean age of the HCW was 47.7 ± 6.5 years and they consisted of eight nurses, four medical doctors, one medical social worker and one health visitor. Ten domains of the TDF were represented among the facilitators and barriers against the recommendation of HPV vaccination by the HCW, except the goals; memory, attention, decision process; emotion; and behavioral regulation domains. The domains with the highest frequency of concepts were: knowledge, skills, social/professional role and identity, beliefs about capabilities, beliefs about consequences, intention and environmental context and resources. Domains with conflicting statements in the concept were: environmental context and resources, and beliefs about consequences. While those with perceived strength of concept were: social influences, reinforcement and optimism.

**Conclusion:**

All the 10 identified domains of the TDF are potential areas of focus for strategies for improving the recommendation of HPV vaccine for adolescents by health care workers in Nigeria and other countries with similar sociocultural settings.

**Supplementary Information:**

The online version contains supplementary material available at 10.1186/s12913-022-08224-7.

## Background

The human Papillomavirus (HPV) vaccination is a primary strategy for the prevention of cervical cancer, one of the leading female specific cancers globally [1]. The vaccine is to be given in early adolescence, before sexual debut for maximum effectiveness [2]. Therefore, the HPV vaccine is scheduled for early adolescence in most countries. While most developed countries have HPV vaccine as a routine vaccine for adolescents, the vaccine is just being introduced in African countries where most cases of cervical cancer are found [3]. Many African countries are able to access the HPV vaccine through the assistance of GAVI, The Vaccine Alliance [4]. Nigeria is currently in the pre-introduction era of the vaccine and it is important to make adequate preparation for the vaccine’s introduction for good uptake among adolescents. Presently, the vaccine is available in the country, being marketed by pharmaceutical companies but only rich families are able to afford it due to the exorbitant cost.

Health care workers (HCW) play strategic roles in guiding parents and adolescents in decision making process about the uptake of health care services [5, 6]. This stems from the trust that both develop overtime with repeated interactions with the HCW while making important health related decisions. Health care workers provide both preventive and curative services and they are a ready source of health information for families. This endears them to the families they provide care for and they are able to influence decisions that families take regarding health issues. Parents have identified HCW as the most frequent source of information about vaccines [7] and African parents have indicated willingness to allow their adolescents to take HPV vaccines if it was introduced by HCW because they trust the source [5, 6]. Recommendations of HPV vaccine for adolescents is one of the services that HCW can provide for families.

There have been varying reports in the literature about HCW’s disposition towards the recommendation of HPV vaccine for adolescents. While most HCW agreed that the vaccine protects against HPV which is responsible for most cases of cervical cancer, their role in the uptake of the HPV vaccine by adolescents can be positive or negative [8]. Studies from some African countries have reported that some HCW do not have adequate knowledge about HPV vaccine [9, 10]. This may make them shy away from discussing the HPV vaccine with families, and when they do, they may give wrong information [11] which could be misleading. In the United States, it was reported that some HCW actively recommend the HPV vaccine for adolescents and have many of the adolescents they see in their services vaccinated [12]. There were also instances in which HCW believed the HPV vaccine can be delayed because of the relationship between its recommended time of administration and the perceived onset of sexual activity by adolescents [13]. This makes them delay the introduction of the HPV vaccine to adolescents that they perceived have low risk of engaging in sexual activities and this sometimes results in non-uptake of the vaccine by adolescents subsequently. However, both HCW and parents of adolescents in a study admitted that it was difficult to predict the timing of onset of sexual activities in adolescents [13], making the delay of HPV vaccination for them to be dangerous. Obviously, this type of HCW disposition to the HPV vaccine contributes to the low uptake of HPV vaccine compared with other vaccines being given in adolescence [14, 15]. It is therefore important to understand the factors that encourage or deter HCW to recommend HPV vaccine for adolescents because this will provide a basis for training and provision of necessary guidance and support to increase their recommendation of the vaccine for adolescents.

The Theoretical Domain Framework (TDF) is a descriptive frame work that was developed by implementation researchers and behavioral scientist [16] initially from 33 behavioral theories that are relevant to implementation and 128 theoretical constructs which are relevant to behavioral change [16, 17]. These were then formulated into 12 domains which cover physical and social environment, as well as individual motivation and capability [17]. The framework was later validated and revised to create 14 domains from 84 constructs [18]. This framework can therefore be used to determine the cognitive, affective, social and environmental influences on behavior [16]. Each of these domains is a mediator for change in behavior and this makes the TDF important in implementation research as it is easy to monitor progress and address any issue that may arise directly. This framework is useful in examining the facilitators and barriers that HCW have in recommending HPV vaccine for adolescents as it will not only identify the associated problems and guide in the design of appropriate interventions to address the problems, but it can also provide the means of monitoring the impact of interventions. There are evidences that behavioral interventions with theoretical base are more effective than those that are not based on theories [19]. This study therefore explored the facilitators and barriers for the recommendation of HPV vaccine for adolescents by HCW who are directly in charge of vaccination in Ibadan, Southwest Nigeria.

## Methods

### Study area

This study was conducted in ward 3 of Ibadan North Local Government Area (LGA) in Southwest Nigeria. This LGA had a projected population of 432,900 in 2016 [20] and it houses both privately owned and government owed health care facilities where HPV vaccines could be obtained. There were 19 primary health centers and one secondary health center in the study area. Ibadan North LGA was also located near a government owned tertiary health facility where HPV vaccine was readily available. One of the primary health centers located in the study area was owned by this tertiary health center.

### Study design

This was an exploratory cross sectional study using maximum variation approach and qualitative data was obtained using Key Informant Interviews (KII).

### Sampling

The HCW who were involved in vaccination were selected purposively using maximum variation approach to ensure that different categories of healthcare workers were represented. The sampling was done across different levels of health care service provision (primary, secondary and tertiary health facilities) as well as job categories (Medicine, nursing, social work and health visitation). The health facilities selected were: two primary health centers (including the one owned by the tertiary health center), the only secondary health center as well as the only tertiary health center. The initial plan was to interview two HCWs for each job categories (pediatricians, family physicians, nurses [two each at the two primary health facilities, secondary and tertiary health care levels], social workers and health visitors).

### Study participants

These were HCW who have been working at the immunization centers in the study area for at least a year. Therefore, the HCWs who were selected for this study were two pediatricians, two family physicians, eight nurses (two each for the two primary health centers, secondary and tertiary health centers). However, only one social worker (at tertiary health facility) who was involved in vaccination was available as at the time of data collection. Also, there was only one health visitor (also at the tertiary health facility) that was eligible to participate in the study. Each HCW was selected with the assistance of the different heads of health care facilities and heads of departments. All the selected HCW agreed to participate in the research.

### Study instrument and data collection

An interview guide was developed based on the researchers’ experiences and previous research reports. The interview guide was designed to explore the extent of the knowledge of the HCW about HPV, HPV vaccine for adolescents, experiences with recommendation of the vaccine for adolescents, specifically the facilitators and barriers for its recommendation. Data was collected by two trained research assistants who had masters degree in Public Health, with one conducting the interview while the other took notes and timed the interview. Neither of the authors participated in the interviews in order to remove bias during the interview process because both were pediatricians with clinical experiences in vaccination procedures. Both were also working at the tertiary health facility where some participants were selected and so, were known by some of the participants. While FMB is a female, OOO is a male. Each interview session took place at conducive locations selected by the HCW at their work places and two audio recorders were used simultaneously for recording the interviews. The biodata of each HCW was taken at the beginning of the interview and each session lasted between 13 to 36 min. Data saturation (no new information was added compared to the content of the preceeding interview) was reached with the eighth interview but all the preselected HCWs were interviewed to achieve the maximum variation sampling.

### Data analysis

Data was analyzed using content analysis by FMB and two research assistants who first studied the TDF to be familiar with it. The data was coded deductively using the domains of the TDF independently. The codes were then reconciled in a common meeting to ensure the same codes were grouped under each domain of the TDF. Where there was disparity in the grouping, all three coders reviewed the code concerned until a consensus was reached. In cases where a code fitted into two or more domains, this was discussed and the code was added to all the relevant domains. Thereafter, specific facilitators and barriers were identified by grouping codes under each domain into core concepts which were identified as facilitator/barrier for HCW’s recommendation of HPV vaccine for adolescents. Each concept was counted once for each participant and there was a frequency count across all interviews [16]. These concepts were described using phrases that best described all the specific responses from the study participants that were relevant to the concept. FMB identified relevant quotes from the data which represented each of these concepts and the other two coders examined the quotes to determine if they correctly represented each concept. The relevant domain of TDF which were relevant to understand the HCW perception of facilitators and barriers for the recommendation of HPV vaccine for adolescents were identified based on criteria used in earlier researches [21, 22]. These were: high frequency of concept (defined as greater than seven for this study, that is, more than half of the number of participants), presence of conflicting statements in the concept and perceived strength of the concept (jointly determined by FMB and OOO based on their expertise and experiences) to affect the recommendation of the vaccine.

### Ethical considerations

The protocol for this study was approved by the University of Ibadan and University College Hospital Institutional Review Board. All HCW gave written informed consent after the study had been explained to them. The names of the HCW were not recorded in the biodata that was collected and in the interviews.

## Results

In all 14 HCW were interviewed and they were made up of eight nurses, four medical doctors, one medical social worker and a home visitor. Their mean age was 47.7 ± 6.5 years. The other details about their demographic are as shown in Table 1. The responses of the HCW to questions about the recommendation of HPV vaccine for adolescents fitted into 10 of the 14 domains of TDF. The domains that were represented are presented here in three groups: capability, motivation and opportunity as reported in earlier literature [16, 18, 21].

**Table 1:** Sociodemographic characteristics of health care workers included in the study

Participants	Age (years)	Gender	Level of healthcare practice	Years of experience
Tertiary health facility nurse	57	Female	Tertiary	27
Pediatrician	46	Male	Tertiary	9
Pediatrician	50	Female	Tertiary	16
Primary health care nurse	36	Female	Primary	6
Secondary health facility nurse	45	Female	Secondary	9
Family Physician	42	Male	Tertiary	5
Family Physician	50	Female	Tertiary	8
Primary health care nurse	55	Female	Primary	23
Health Visitor	56	Female	Tertiary	20
Social Worker	52	Female	Tertiary	11
Secondary health facility nurse	44	Female	Secondary	3
Primary health care nurse	39	Female	Primary	14
Tertiary health facility nurse	44	Female	Tertiary	4
Primary health care nurse	53	Female	Primary	20

### Key concepts within the domains of TDF

The knowledge and skills domain constituted the *capability* group of facilitators and barriers for the recommendation of HPV vaccine for adolescents. Regarding the knowledge domain, almost all the HCW had knowledge about the HPV vaccine and the females had more detailed knowledge about the different types of HPV vaccines, as well as their doses. Some nurses from the PHCs had little or no knowledge about the vaccine as shown in Table 2. For the skill domain, half of the HCW talked about the communication skills that they possessed which were being used while recommending HPV vaccine.

**Table 2:** Facilitators and barriers of Health care workers’ capability to recommend HPV vaccine using the TDF^a^

Domains	Specific facilitator/barrier	Sample quotes	Frequency out of 14
Knowledge	Correct knowledge (F)	‘Vaccination…between the ages of 9 and 26…after the age of 26, people still develop immunogenicity’ *Family Physician 1, Tertiary HF*	12
	Incorrect knowledge (B)	‘I think it can be given to those that are not sexually active’ *Nurse 1, Primary HC*	1
	No knowledge (B)	‘I don’t know the content of the vaccine…I don’t know all those things’ *Nurse 3, Primary HC*	1
Skills	Communication skills with clients (F)	‘we know how to explain it to them in the language they understand' *Nurse 3, Primary HC*	7
	Training for HPV vaccine administration (F)	‘we have had several summits…’ *Nurse 8, Tertiary HF*	1
	Precautions before vaccine uptake (F)	‘if they are exposed…one can screen for HPV…and get vaccinated’ *Family Physician 1, Tertiary HF*	1

^a^Theoretical Domains Framework. *F* Facilitator, *B* Barrier, *HC* Health facility

While some spoke about the trainings that were being organized to help HCW to develop skills in the recommendation of HPV vaccine for adolescents.

*Motivation* group consisted of Social/professional role and identity, Beliefs about capabilities, Optimism, Beliefs about consequences, Reinforcement and Intention domains as shown in Table 3. Most of the HCW believed that it was their responsibility as health care professionals to recommend HPV vaccine for adolescents and two believed that their profession also motivates them to learn new things (including the principles behind the recommendation of HPV vaccine) through reading. While many believed that they could recommend the vaccine for adolescents, a nurse thought otherwise, because she did not have the required knowledge to do so. Optimism about the recommendation of the vaccine was seen in the responses of only two HCW and they believed that their continued recommendation of the vaccine will eventually lead to the acceptance of the vaccine for adolescents. All the HCW had positive belief about the recommendation of HPV vaccine for adolescents but some of them still felt that it could be a trigger for adolescents to indulge in indiscriminate sex. However, a nurse believed that the recommendation process usually comes with responsible sex education and that the process will actually curtail indiscriminate sex as well as improve the knowledge of the communities about the vaccine. The other consequence for the recommendation was the possibility of allergic reaction. Only the physicians mentioned things that can reinforce the recommendation of HPV vaccines by HCW. All the HCW except a nurse from a PHC had the intention to recommend HPV vaccine for adolescents freely. The nurse hinged her recommendation of the HPV vaccine to getting adequate knowledge about the vaccine. The other details about the specific facilitators/barriers in the reinforcement and intention domains are as shown in Table 3.

**Table 3:** Facilitators and barriers of Health care workers’ motivation to recommend HPV vaccine using the TDF^a^

Domains	Specific facilitator/Barrier	Sample quotes	Frequency out of 14
Social/professional role and identity	Professional responsibility (F)	‘I am a public health nurse, I give health talk to mothers’ Nurse 2, Tertiary HF	6
	Impetus for learning (F)	I work in a hospital…I want to learn more…I went through the training’ *Social worker, Tertiary HF*	2
Beliefs about capabilities	Ability to recommend HPV vaccine and ensure uptake (F)	‘most people I have recommended it to, they have taken it’ *Family Physician 1, Tertiary HF*	8
	Cannot recommend HPV vaccine (B)	‘no, if the knowledge is…adequate, maybe I will recommend it’ *Nurse 1, PHC*	1
Optimism	Recommendation of HPV vaccine will lead to its uptake (F)	‘and even with our own effort to health educate them, I know they will turn up’ *Nurse 4, Secondary HF*	2
Beliefs about consequences	Reduce cervical and other HPV associated cancers’ burden (F)	‘…reduce the incidence of cervical cancer…cut down maternal mortality…would increase life expectancy’ *Pediatrician 1, Tertiary HF*	13
	Increased indiscriminate sex (B)	‘they will go on with this false sense of security that they are protected against HPV, they are having indiscriminate sexual exposure’ *Family Physician 1, Tertiary HF*	2
	Adverse reactions (B)	‘…watch that there is no allergy’ Family *Physician 2, Tertiary HF*	1
	Reduced promiscuity (F)	‘It will caution some ladies from being promiscuous’ *Nurse 6, Secondary HF*	1
	Improved community knowledge of HPV vaccine (F)	‘…it will also make the community to be aware of what the vaccine is about’ *Nurse 1, PHC*	1
Reinforcement	Structured adolescent health program (F)	‘…establish a well-structured adolescent program…how you will reach the people that are the recipients of the vaccine…’ *Pediatrician 1, Tertiary HF*	1
	HPV vaccine funding from health insurance (F)	‘Government can…put it under NHIS…out of pocket payment will not be there’ Family *Physician 2, Tertiary HF*	1
	Inclusion in routine vaccine schedule (F)	‘…including it in the NPI…it gives a mother a sense of obligation that I need to vaccinate my child’ *Family Physician 1, Tertiary HF*	1
Intention	Intention to recommend HPV vaccine freely (F)	‘I will recommend it gladly’ *Medical Social worker, Tertiary HF*	13
	Intention to recommend when knowledge is adequate (F)	‘if I have the knowledge now, I will know who it is for, the side effects, the mode of action and all those things… will make me to know who to recommend it’ *Nurse 3, PHC*	1

^a^Theoretical Domains Framework. F Facilitator, B Barrier, HC Health facility

The *opportunity* group was made up of social influences and environmental context and resources as shown in Table 4. Some HCW have had opportunities to recommend the HPV vaccine for adolescents through interpersonal relationships and involvement in social groups. However, some traditional beliefs about cancers were identified as barriers for the recommendation of the vaccine. Other barriers in the environmental context and resources include vague government policies about HPV vaccine for adolescents and the high cost of the vaccine. However, the identified facilitators were inclusion of the vaccine in the national health insurance scheme to stem out of pocket payment and inclusion in the routine vaccination schedule so that parents will remember to vaccinate their adolescents.

**Table 4:** Facilitators and barriers of Health care workers’ opportunity to recommend HPV vaccine using the TDF^a^

Domain	Specific facilitator/Barrier	Sample Quote	Frequency out of 14
Social influences	Interpersonal relationships (F)	‘personal relationship…with a particular family’ *Pediatrician 1, Tertiary HF*	2
	Involvement in social groups (F)	‘I have a youth program…I have spoken to youths about it’ *Medical Social worker, Tertiary HF*	2
	Traditional beliefs about cancers (B)	‘People believe that cancer is a punishment from God…it is a spiritual problem so vaccine can never be a solution’ *Nurse 1, Primary HC*	2
Environmental context and resources	High cost of HPV vaccine (B)	‘it’s like it’s for elites…the price is high’ *Health visitor, Tertiary HF*	11
	Vague Government/ hospital policy (B)	‘whether it is now a full policy by the federal government…I cannot say for now’ *Family Physician 2, Tertiary HF*	3
	Availability and storage of HPV vaccine (B)	‘…availability has been a challenge’ *Family Physician 1, Tertiary HF*	2
	Ignorance, rumors among the populace (B)	‘it is all the rumors and all the misconceptions about the vaccines, people have said it is a license to become promiscuous’ *Pediatrician 2, Tertiary HF*	5
	Parents’ low risk perception about adolescents’ sexual activities (B)	‘some believe that my girl is not promiscuous now, so why would I?’ *Family Physician 1, Tertiary HF*	4

^a^Theoretical Domains Framework. *F* Facilitator, *B* Barrier, *HC* Health facility

### Domains of TDF that were relevant

Based on high frequency of occurrence, the relevant domains are: knowledge, skills, social/professional role and identity, beliefs about capabilities, beliefs about consequences, intention and environmental context and resources. Those that are relevant based on conflicting statements in the concept are: environmental context and resources, and beliefs about consequences. While those that are relevant due to the perceived strength of the concept are: social influences, reinforcement and optimism. Social influences were considered relevant because the HCW build social relationships with the families and communities they work with and the recommendation of the HPV vaccine will be for the adolescents from these families and communities. So, though most of the HCW interviewed did not really mention the social influences, they are important. Also, reinforcement is required in the policies and action of the government and the HCW if HPV vaccine recommendation is to become a routine behavior for HCW. Optimism is also an important motivation which will encourage HCW to recommend HPV vaccine. Therefore, all the 10 identified domains were considered relevant among the studied HCW.

## Discussion

The HPV vaccine is not yet included in the routine vaccination schedule in Nigeria but most of the HCW in this study were familiar with the vaccine and have been recommending it. They were able to provide useful insights into the facilitators and barriers that they encounter while recommending the HPV vaccine for adolescents in their practice and these were presented using the domains of TDF. Knowledge, belief about consequences and intention were the domains of the TDF with the highest frequency of concepts about the facilitators and barriers that these HCW encounter while recommending HPV vaccine for adolescents.

Knowledge and skill are prerequisite for carrying out any procedure and both of these determine the capability of an individual to recommend the HPV vaccine in the first place. Without these two, it is absolutely impossible for any HCW to appropriately recommend HPV vaccine. It was obvious that the HCW have been improving their knowledge and skills through self help, aside from the basic skills that they already had about presenting vaccines to parents. This could explain the varying level of knowledge and skills that they had about the recommendation of HPV vaccine. Also, Osazuwa-Peters et al. reported women’s better knowledge about HPV compared with men in a study from the United States [23] just as seen among these HCW. This may imply the attention that the female HCW pay to the vaccine because cervical cancer is a female specific cancer and the female HCW are also prone to developing it. Earlier research in Nigeria and other African countries have shown that HCW have good knowledge of the HPV vaccine [24] but their skills in recommending this vaccine is under researched unlike their counterparts in developed countries [15, 25]. The process of recommendation of the HPV vaccine have been shown to be important in parents’ decision making about their adolescents as this determines if the vaccine will be taken, deferred or rejected [26]. The introduction of vaccines in countries is normally preceded by training of HCW to improve their knowledge about the vaccine and this can bridge the gap for the HCW who did not have adequate knowledge, as well as those with the incorrect knowledge and skills to recommend HPV vaccine for adolescents. More attention however needs to be paid to the skills required to present this vaccine to parents and adolescents. When HCW have both the knowledge and skills to recommend HPV vaccine, the intention to recommend the vaccine will definitely improve [8].

The motivation of HCW is required in the successful recommendation of HPV vaccine for adolescents as it will be a thrust to ensure that the process is carried out correctly and more attention is paid to parents who have difficulties in making decisions about the vaccine for their adolescents. It is therefore a good observation that many of the HCW interviewed already perceived the recommendation of HPV vaccine as part of their responsibilities because this will make them to take the vaccine as an important one. This may be the drive for the self development that many of them already embarked on to get knowledge and skills to recommend the vaccine. This will most likely impact on the beliefs about their capabilities [8] as HCW to recommend this vaccine and boost their optimism that their continued recommendation will eventually lead to improvement in the uptake of the vaccine. However, those with inadequate knowledge of the HPV vaccine believed they could not recommend the vaccine for adolescents. This still underscores the importance of adequate knowledge and skill of HCW to recommend the vaccine.

Most beliefs about the consequences of the recommendation of HPV vaccine for adolescents were positive with the most common being the reduction in the cases of cervical cancer. It has been shown that countries that have introduced the vaccine have started recording reduction in the incidence of cervical cancer [27, 28]. This will be a welcome development when this vaccine becomes routine in Nigeria as the over 9,000 women’s deaths attributable to cervical cancer in the country will be overturned [29]. However, it is important to continue to stress that the vaccine can also protect against other cancers like head, neck, anal and penile cancers [30, 31]. These other cancers are commoner in males while the penile cancers are male specific [31]. This will make HCW and parents appreciate the need to also vaccinate male adolescents as well. It has been shown that the vaccination of both gender is cost effective if the burden of all HPV related cancers is considered [32]. However, the belief that the vaccine will give adolescents a false sense of security from other sexually transmitted diseases by engaging in indiscriminate sex was expressed by some of the HCW just as seen in earlier research [33, 34]. If this notion is not corrected with proofs, it may hinder the HCW from freely recommending the vaccine. Earlier studies have shown that the administration of the vaccine did not increase sexual activities of adolescents [35]. In contrast, there was a report of delay in the initiation of sexual activities among adolescents who received HPV vaccine similar to the belief of one of the HCW that the recommendation of the vaccine can be accompanied with the promotion of responsible sexuality education [36]. This type of pre-vaccination counselling may not be currently practiced by many of the HCW in this study as discussions about sex are still shrouded in secrecy in many African communities. Earlier research has also reported that HCW who find it difficult to talk about sex were less likely to discuss HPV vaccine with parents of adolescents [15].

One of the requirements for GAVI’s support to help developing countries to access subsidized HPV vaccines for adolescents is that the countries must be able to demonstrate the ability to deliver repeat doses of vaccine to adolescents. Most African countries that have introduced HPV vaccination in their routine vaccination schedule have adopted mainly school based vaccination strategy [37, 38]. This may be impossible in Nigeria as many of the country’s adolescents are out of school and adolescent health program is currently not well developed in the country [39]. It is therefore pertinent that a structure to reach adolescents effectively should be put in place to ensure a wide coverage of the HPV vaccine when it is eventually introduced. This may be a community based program which will provide access for both in and out of school adolescents. It is important that all stakeholders start thinking about the appropriate approach to reach adolescents with the HPV vaccine. It was not surprising that the high cost of HPV vaccine was stated as a barrier to recommending the vaccine as this has been a major concern to all stakeholders as seen in earlier research [40, 41]. The inclusion of the vaccine under the National Health Insurance scheme as suggested by some HCW will not only remove the burden of out of pocket payment from the parents, but HCW will be encouraged recommend the vaccine readily when necessary. Also, the inclusion of the vaccine in the routine vaccination schedule would be an offshoot of government policy and each hospital managementboard will adopt this policy. This will remove the ambiguity in the policy about the vaccine and give the HCW the confidence to recommend HPV vaccine for adolescents.

There were also facilitators and barriers against the opportunities to recommend the HPV vaccine by the HCW. The relationship between many HCW and families of adolescents is based on trust and this explains the reason why consistently, HCW have remained a major influence for parents to allow their adolescents to have the HPV vaccine. The HCW also have this influence outside the clinic in their various social groups and they can leverage on their influence to recommend the vaccine as well. This is because the same trust that parents and adolescent have in them in the clinic also exist in such circles. However, there were more barriers for the recommendation of HPV vaccine under the environmental concept and resources domain and more effort is required to address these barriers. Suggestions about how the high cost of the HPV vaccine and the vague policies about HPV vaccine can be addressed have been discussed earlier. The low risk perception by parents about their adolescents’ sexual activities has been reported in the literature [13]. Improving the skills of parents to understand how the vaccine works can allay their fears and encourage them not delay eligible adolescents from getting the vaccine. The HCW had real fears about the availability of optimal storage facilities for the vaccine, but, there have been a general improvement in the cold chain facilities available in Nigeria in the last decade [42]. This will help to ensure optimal storage conditions for the HPV vaccine when it becomes routine. The country was able to surmount the apathy toward the uptake of polio vaccine which was as a result of rumors and suspicions about the vaccine. The strategies employed to address the problem which resulted in Nigeria becoming polio free can also be used to ensure the acceptance of the HPV vaccine.

## Conclusion

This study was able to explore the facilitators and barriers that HCW in charge of vaccination experienced in the recommendation of the HPV vaccine for adolescents using the TDF. While the knowledge, belief about consequences and intention domains had the highest frequencies of concept about the barriers and facilitators for recommending HPV vaccine, each of the identified relevant domains are potentially relevant in changing how HCW recommend HPV vaccine for adolescents. This is because of the conflicting concepts that were seen in some domains and the perceived strength of some other domains in influencing the recommendation of the vaccine. To the best of our knowledge, this is the first study in our region to explore the facilitators and barriers for the recommendation of HPV vaccine for adolescents by HCW using TDR. This offers a baseline to develop strategies to improve the capacity of HCW to recommend the vaccine in Nigeria and in countries with similar settings.

## Supplementary information

**Additional file 1.**

**Additional file 2.**

**Additional file 3.**

**Additional file 4.**

**Additional file 5.**

**Additional file 6.**

**Additional file 7.**

**Additional file 8.**

**Additional file 9.**

**Additional file 10.**

**Additional file 11.**

**Additional file 12.**

**Additional file 13.**

**Additional file 14.**

**Additional file 15.**

## Data Availability

The datasets generated or analyzed during this study are included in this published article as supplementary information files.

## Abbreviations

HPV: Human Papillomavirus vaccine
HCW: Health care worker
TDF: Theoretical Domain Framework
PHC: Primary Health Center
HF: Health facility

## References

[1] Fitzmaurice C, Dicker D, Pain A, Hamavid H, Moradi-Lakeh M, MacIntyre MF, Allen C, Hansen G, Woodbrook R, Wolfe C (2015). The Global Burden of Cancer 2013. JAMA Oncol.

[2] Gertig DM, Brotherton JM, Budd AC, Drennan K, Chappell G, Saville AM (2013). Impact of a population-based HPV vaccination program on cervical abnormalities: a data linkage study. BMC Med.

[3] Abdullahi LH, Hussey GD, Wiysonge CS, Kagina BM: Lessons learnt during the national introduction of human papillomavirus (HPV) vaccination programmes in 6 African countries: Stakeholders' perspectives. South African medical journal = Suid-Afrikaanse tydskrif vir geneeskunde*.* 2020;110(6):525–531. 10.7196/SAMJ.2020.v110i6.14332 32880566

[4] Cagney H.: GAVI to fund HPV vaccines in low-income countries. The Lancet Oncology*.* 2013;14(3):E92. 10.1016/s1470-2045(13)70050-0 23580961

[5] Getrich CM, Broidy LM, Kleymann E, Helitzer DL, Kong AS, Sussman AL (2014). Different models of HPV vaccine decision-making among adolescent girls, parents, and health-care clinicians in New Mexico. Ethn Health.

[6] Pell C, Straus L, Andrew EV, Menaca A, Pool R (2011). Social and cultural factors affecting uptake of interventions for malaria in pregnancy in Africa: a systematic review of the qualitative research. PLoS ONE.

[7] Omer SB, Orenstein WA, Koplan JP (2013). Go big and go fast–vaccine refusal and disease eradication. N Engl J Med.

[8] Lutringer-Magnin D, Kalecinski J, Barone G, Leocmach Y, Regnier V, Jacquard AC, Soubeyrand B, Vanhems P, Chauvin F (2011). C L: **Human papillomavirus (HPV) vaccination: Perception and practice among French general practioners in the year since licensing**. Vaccine.

[9] Makwe CC, Anorlu RI. Knowledge of and attitude toward human papillomavirus infection and vaccines among female nurses at a tertiary hospital in Nigeria. Int J Women’s Health. 2011;3:313–7. 10.2147/IJWH.S22792 PMC3181212 21976985

[10] Anorlu RI, Orakwue CO, Oyeneyin L, Abudu OO (2004). Late presentation of patients with cervical cancer to a tertiary hospital in Lagos: what is responsible?. Eur J Gynaecol Oncol.

[11] Arwanire EM, Mbabazi W, Mugyenyi P (2015). Communicating vaccine safety in the context of immunization programs in low resource settings. Curr Drug Saf.

[12] Allison MA, Hurley LP, Markowitz L, Crane LA, Brtnikova M, Beaty BL, Snow M, Cory J, Stokley S, Roark J, et al. Primary Care Physicians’ Perspectives About HPV Vaccine. Pediatrics. 2016;137(2): e20152488. 10.1542/peds.2015-2488 PMC6104512 26729738

[13] Perkins RB, Clark JA, Apte G, Vercruysse JL, Sumner JJ, Wall-Haas CL, Rosenquist AW, Pierre-Joseph N (2014). Missed opportunities for HPV vaccination in adolescent girls: a qualitative study. Pediatrics.

[14] Soon R, Dela Cruz MR, Tsark JU, Chen JJ, Braun KL. A Survey of Physicians’ Attitudes and Practices about the Human Papillomavirus (HPV) Vaccine in Hawaii. Hawai’i journal of medicine & public health : a journal of Asia Pacific Medicine & Public Health. 2015;74(7):234–41. PMC4507363 26225269

[15] Gilkey MB, McRee AL (2016). Provider communication about HPV vaccination: A systematic review. Hum Vaccin Immunother.

[16] Atkins L, Francis J, Islam R, O’Connor D, Patey A, Ivers N, Foy R, Duncan EM, Colquhoun H, Grimshaw JM, et al. A guide to using the Theoretical Domains Framework of behaviour change to investigate implementation problems. Implementation science : IS. 2017;12(1):77. 10.1186/s13012-017-0605-9 PMC5480145 28637486

[17] Francis JJ, O’Connor D, Curran J. Theories of behaviour change synthesised into a set of theoretical groupings: introducing a thematic series on the theoretical domains framework. Implementation science : IS. 2012;7:35. 10.1186/1748-5908-7-35 PMC3444902 22531601

[18] Cane J, O’Connor D, Michie S. Validation of the theoretical domains framework for use in behaviour change and implementation research. Implementation science : IS. 2012;7:37. 10.1186/1748-5908-7-37 PMC3483008 22530986

[19] Glanz K, Bishop DB (2010). The Role of Behavioral Science Theory in Development and Implementation of Public Health Interventions. Annu Rev Public Health.

[20] City Population: Oyo State in Nigeria. In*.*, March 21, 2016 edn; 2016.

[21] Patey AM, Islam R, Francis JJ, Bryson GL, Grimshaw JM (2012). the Canada PPT: Anesthesiologists’ and surgeons’ perceptions about routine pre-operative testing in low-risk patients: application of the Theoretical Domains Framework (TDF) to identify factors that influence physicians’ decisions to order pre-operative tests. Implement Sci.

[22] Islam R, Tinmouth AT, Francis JJ, Brehaut JC, Born J, Stockton C, Stanworth SJ, Eccles MP, Cuthbertson BH, Hyde C, et al. A cross-country comparison of intensive care physicians’ beliefs about their transfusion behaviour: a qualitative study using the Theoretical Domains Framework. Implementation science : IS. 2012;7:93. 10.1186/1748-5908-7-93 PMC3527303 22999460

[23] Osazuwa-Peters N, Adjei Boakye E, Mohammed KA, Tobo BB, Geneus CJ, Schootman M. Not just a woman’s business! Understanding men and women’s knowledge of HPV, the HPV vaccine, and HPV-associated cancers. Prev Med. 2017;99:299–304. 10.1016/j.ypmed.2017.03.014 28341458

[24] Awodele O, Adeyomoye AA, Awodele DF, Kwashi V, Awodele IO, Dolapo DC (2011). A study on cervical cancer screening amongst nurses in Lagos University Teaching Hospital, Lagos. Nigeria J Cancer Educ.

[25] Dempsey AF, Abraham LM, Dalton V, Ruffin M: Understanding the reasons why mothers do or do not have their adolescent daughters vaccinated against human papillomavirus. Ann Epidemiol*.* 2009;19(8):531-8. 10.1016/j.annepidem.2009.03.011 PMC2880849 19394865

[26] Kempe A, O'Leary ST, Markowitz LE, Crane LA, Hurley LP, Brtnikova M, Beaty BL, Meites E, Stokley S, Lindley MC: HPV Vaccine Delivery Practices by Primary Care Physicians. Pediatrics*.* 2019;144(4):e20191475. 10.1542/peds.2019-1475 PMC8297056 31527175

[27] Hariri S, Bennett NM, Niccolai LM, Schafer S, Park IU, Bloch KC, Unger ER, Whitney E, Julian P, Scahill MW (2015). Reduction in HPV 16/18-associated high grade cervical lesions following HPV vaccine introduction in the United States - 2008–2012. Vaccine.

[28] Garland SM, Kjaer SK, Munoz N, Block SL, Brown DR, DiNubile MJ, Lindsay BR, Kuter BJ, Perez G, Dominiak-Felden G, et al. Impact and Effectiveness of the Quadrivalent Human Papillomavirus Vaccine: A Systematic Review of 10 Years of Real-world Experience. Clinical infectious diseases : an official publication of the Infectious Diseases Society of America. 2016;63(4):519–27. 10.1093/cid/ciw354 PMC4967609 27230391

[29] Ferlay J, Shin H-R, Bray F, Forman D, Mathers C, Parkin DM: Estimates of worldwide burden of cancer in 2008: GLOBOCAN 2008. 2010;127(12):2893–2917. 10.1002/ijc.25516 21351269

[30] van Bogaert L (2013). Are the currently existing anti-human papillomavirus vaccines appropriate for the developing world?. Ann Med Health Sci Res.

[31] Canepa P, Orsi A, Martini M, Icardi G (2013). HPV related diseases in males: a heavy vaccine-preventable burden. J Prev Med Hyg.

[32] Kotsopoulos N, Connolly MP, Remy V (2015). Quantifying the broader economic consequences of quadrivalent human papillomavirus (HPV) vaccination in Germany applying a government perspective framework. Heal Econ Rev.

[33] Krakow MM, Jensen JD, Carcioppolo N, Weaver J, Liu M, Guntzviller LM. Psychosocial predictors of human papillomavirus vaccination intentions for young women 18 to 26: religiosity, morality, promiscuity, and cancer worry. Women’s health issues : official publication of the Jacobs Institute of Women’s Health. 2015;25(2):105–11. 10.1016/j.whi.2014.11.006 25648488

[34] Mupandawana ET, Cross R (2016). Attitudes towards human papillomavirus vaccination among African parents in a city in the north of England: a qualitative study. Reprod Health.

[35] Smith LM, Kaufman JS, Strumpf EC, Levesque LE: Effect of human papillomavirus (HPV) vaccination on clinical indicators of sexual behaviour among adolescent girls: the Ontario Grade 8 HPV Vaccine Cohort Study. CMAJ : Canadian Medical Association journal = journal de l'Association medicale canadienne*.* 2015;187(2):E74-e81. 10.1503/cmaj.140900 PMC4312170 25487660

[36] Oldach BR, Katz ML (2012). Ohio Appalachia public health department personnel: human papillomavirus (HPV) vaccine availability, and acceptance and concerns among parents of male and female adolescents. J Community Health.

[37] Torres-Rueda S, Rulisa S, Burchett HE, Mivumbi NV, Mounier-Jack S. HPV vaccine introduction in Rwanda: Impacts on the broader health system. Sexual & reproductive healthcare : official journal of the Swedish Association of Midwives. 2016;7:46–51. 10.1016/j.srhc.2015.11.006 26826045

[38] Turiho AK, Okello ES, Muhwezi WW, Katahoire AR (2017). Perceptions of human papillomavirus vaccination of adolescent schoolgirls in western Uganda and their implications for acceptability of HPV vaccination: a qualitative study. BMC Res Notes.

[39] Umeh IB, Nduka SO, Ekwunife OI. Mothers’ willingness to pay for HPV vaccines in Anambra state, Nigeria: a cross sectional contingent valuation study. Cost effectiveness and resource allocation : C/E. 2016;14:8. 10.1186/s12962-016-0057-0 PMC4895896 27274335

[40] Holman DM, Benard V, Roland KB, Watson M, Liddon N, Stokley S (2014). Barriers to human papillomavirus vaccination among US adolescents: a systematic review of the literature. JAMA Pediatr.

[41] Asiedu GB, Breitkopf CR, Kremers WK, Ngo QV, Nguyen NV, Barenberg BJ, Tran VD, Dinh TA. Vietnamese Health Care Providers’ Preferences Regarding Recommendation of HPV Vaccines. Asian Pacific journal of cancer prevention : APJCP. 2015;16(12):4895–900. 10.7314/apjcp.2015.16.12.4895 26163611

[42] Sarley D, Mahmud M, Idris J, Osunkiyesi M, Dibosa-Osadolor O, Okebukola P, Wiwa O (2017). Transforming vaccines supply chains in Nigeria. Vaccine.

