# Supplementary material for: Facilitators and barriers of healthcare workers’ recommendation of HPV vaccine for adolescents in Nigeria: views through the lens of theoretical domains framework
Source: BMC Health Serv Res. 2022 Jun 25;22:824. doi: 10.1186/s12913-022-08224-7 (PMC9233785; doi:10.1186/s12913-022-08224-7)
Supplement: Supplementary file 1 — Additional file 1. [file 12913_2022_8224_MOESM1_ESM.docx]

**INTERVIEW ID: 170217_002**

**TYPE OF INTERVIEW: IDI**

**PARTICIPANT: CONSULTANT PAEDIATRICIAN, INSTITUTE OF CHILD HEALTH**

**NUMBER OF PARTICIPANT: 1**

**INTERVIEWER: T**

**TIME OF INTERVIEW: 13: 05**

**LANGUAGE OF INTERVIEW: ENGLISH**

**VENUE OF INTERVIEW: ICH, IBADAN**

**AGE OF PARTICPANT: 50+**

**GENDER: MALE**

**DATE OF INTERVIEW: 17-02-2017**

I: Good morning sir, my name is XXXX and I am here to find out your views, your knowledge and your understanding about cervical cancer, Human papilloma virus ,and Human papilloma virus vaccine, and before I continue this interview, I will like to ask your permission to go on with this interview , do I have your permission to go on with this interview sir

R: yes you do

I: I will also like to get your consent to record this conversation, do I have that sir

R: it’s okay

I: thank you sir, and I want to assure you that everything you are going

R: what is the difference between permission and consent,

I: consent to take the interview and consent to record the conversation, people may not want their conversations audiotaped

R:okay, let’s go, the consent is for the interview, the permission is for the recording

I: yes, that is it, thank you sir, I want to assure you that everything you will be saying is strictly confidential and will be used for this research only

R: so it will not be taken against me in the court or law

I: it will not sir, it will not

R: okay,

I: I will like to know a bit about you, your designation, and how long you have been working

R:my designation, [ what you work as,] I work as a senior research fellow, which is equivalent to senior lecturer, so I have been working since 2008,

I: that’s about 10 years now, next year makes it 10 years, okay sir, what do you know about cervical cancer

R: as the name suggest , it’s a disease of the cervix, and em, cancers generally are cells that are multiplying rapidly more than usual and as a result they outgrow other normal cells and when this happens in the cervix, we call it cervical cancer, and as such it could cause a lot of trouble, {sir do you know any } may be bleeding especially post coital, that is during sex, I mean during and after, it could also, just like any other cancer cause anaemia. They have anaemia, short of blood ,they also are unable to eat well

I: sir, how can this cancer be prevented

R: cervical cancer, well, it is preventable to a reasonable extent, the very major step that is known now and that has been well established is early screening through the use of pap smear, and the aim is if you pick it at the early stages when it is just showing the signs of occurring, the surgeons, can go there, either remove the cells or they can find a way of cauterizing, there is something we call cauterization, you just make the cell dead and because the cervix is easily accessible , this makes the procedure very easy and it’s cheap, the other way to prevent it, which is still within reach is the use of human papilloma virus vaccine, the essence for, the reason for using this vaccine is because the most common factor for ca cervix and a few other cancers is human papilloma virus, so evidence has shown that if you are able to protect women, as early as possible in life against this virus, you can reduce the risk of cervical cancer

I: you mentioned as early as possible, when is that

R: as early as pre adolescence,

I: okay, sir, have you had any training related to cervical cancer

R: I am a medical doctor, so my training has been right from medical school, but in terms of having a special workshop, no,

I: sir, please can you tell me about the human papilloma virus, what exactly is it

R: the virus itself, you want me to go scientific, [ yes sir] I know, I am not sure now, whether it’s a DNA or an RNA virus but what is peculiar about it is that, it has predilection for certain cells that are located in the cervix and also somewhere around the throat, that is the virus

I: the vaccine sir

R: the vaccine, like any other vaccine, these are preparations made through proven scientific method, they are made to look like the virus itself, they are not the virus, they look like antigens and they work against the virus

I; sir, do you know the type of vaccine

R: I am not sure

I: what’s the importance of the vaccine in Nigeria

R:just like any other vaccine, its important, because Ca cervix is now a big health challenge for many countries whether developing or developed countries, so it is important because the vaccines are given to women because you are able to reduce the incidence of cervical cancer, you cut down maternal mortality and generally would increase life expectancy ,

I: sir do you know the recommendation for the vaccine in Nigeria

R: the recommendation, is there a policy already, I am not sure, I am not sure whether there is already any published policy, we know that they talk about it and the ministry of health is also aware but it has not been entrenched in our routine immunization program

I: sir what do you think will be the benefit, if it is introduced into the routine immunization schedule

R: well, it will be of great benefit just like any other vaccine, however, the starting point for HPV, is to first of all establish a well-structured adolescent program, because the question of how you will reach the people that are the recipients of the vaccine is also there and this has not been properly done, until Nigeria is able to put a structure in place and an average adolescent knows that they have access to healthcare, where they can access it , how they can access it, then it won’t work

I: so if we don’t have that we can’t really

R: okay, we can’t build nothing on nothing, you can’t build something on nothing, that’s what I mean for instance, the routine vaccination for infants is predicated on the fact that we have immunization clinics all around or infants welfare clinic as it used to be and several other things have been built on that, but for the adolescent age group, for the grown up children, there is no such arrangement for now

I: so it will be difficult to

R: it is not as if it’s totally impossible, people have talked about even taking it to schools for those children to be reached, but then that has it’s own challenges because the vaccine is not oral, to manage such in school settings is another problem,

I: apart from these challenges you mentioned do you think there are other challenges that may come up,

R: well, one major one is cost,

I: cost of the vaccine

R: yes

I: why do you say that sir

R: well on the average, Nigerians don’t want to pay for anything,[laughter] the routine vaccine , do you think we will have the level of coverage we have even though it is free,bu

I: the cost

R: cost is a major one, and then, the other one is political will, generally I am not sure that our government will be ready to be committed to such an extent that will make it a success, but all these challenges can be overcome if we, if all the stakeholders will come together to sort it

I: so who are the stake holders

R: the stakeholders, the stakeholders will not just be the government alone because every time we are fond of talking about the government alone, the NGOs, the parents themselves, the adults of this nation, of course we can’t call everybody to a meeting, there are community representations all over the place, we talk about vaccine, why is it necessary and all of that, the health workers, because if the health workers don’t believe in the efficacy of the vaccine and the need for it, it cannot be prevented,

I:sir, is there any reason why you will not recommend this vaccine to adolescents

R: why not, I will

I: have you had a cause to recommend the vaccine

R: yes, but a few, on few occasions,

I: can you share one of those

R: well I can only remember once or twice, personal relationship may be with a particular family,

I: but you have not maybe in the course of your duty

R: no, no, no, because in recent years, I rarely see such category of people, [okay] I see very sick children in children emergency ward

I: thank you very much for your time sir, I don’t know if you have anything to add that may help this study

R: well, that may help the study

I: yes

R: I can’t remember the what the study is all about

I: the study is about knowledge and acceptance of human papilloma virus vaccine, we want to know if you know about cervical cancer first,

R: ehm, you have told me all of that, but the purpose

I: of the study

R: when you know the knowledge, when you know the acceptability, so what

I: it will help in seeing what we can do to ensure that the vaccine is

R: collecting this kind of data is good, because it can help in formulating policies, please can I pick this call?

I: yes, you can sir, thank you for your time.
