# Supplementary material for: Facilitators and barriers of healthcare workers’ recommendation of HPV vaccine for adolescents in Nigeria: views through the lens of theoretical domains framework
Source: BMC Health Serv Res. 2022 Jun 25;22:824. doi: 10.1186/s12913-022-08224-7 (PMC9233785; doi:10.1186/s12913-022-08224-7)
Supplement: Supplementary file 2 — Additional file 2. [file 12913_2022_8224_MOESM2_ESM.docx]

**INTERVIEW ID:**

**TYPE OF INTERVIEW: IDI**

**PARTICIPANT: ASSISTANT DIRECTOR, PUBLIC HEALTH NURSING, UNIVERSITY COLLEGE HOSPITAL, IBADAN**

**NUMBER OF PARTICIPANT: 1**

**INTERVIEWER: T**

**TIME OF INTERVIEW: 27:53**

**LANGUAGE OF INTERVIEW: ENGLISH**

**VENUE OF INTERVIEW: UCH, IBADAN**

**AGE OF PARTICPANT: 57**

**GENDER: FEMALE**

**DATE OF INTERVIEW: -02-2017**

I: thank you very much ma, my name is XXXXX and I am here to find out your understanding about cervical cancer, HPV, HPV Vaccine, I want to know what you know, your knowledge as a health care worker, and the interview is not going to take too much of your time, and every information you give will be kept confidential, I need to ask your permission to go on with this interview, I mean your consent to go on with this interview, thank you very much ma, please ma, I will like you to please speak up so that the recorder can pick your voice, please ma, can you tell me a bit about yourself, your designation, how long you have been working as a health worker and some other things about you

R: my names

I: no you don’t need to mention your names

R: I am an assistant director, public health nursing, I have been working in this unit since 1990, and am, I am the head of the immunization, and in this clinic, we give both local and international vaccines

I: how old are you ma

R: I am 57

I: thank you very much ma, ma, I will like to know, can you tell me, what you know about cervical cancer

R: cervical cancer and the human papilloma virus, let’s take it together, they are affecting women, many women worldwide, and ehm, every 2 minutes they say, a woman dies of cervical cancer and it is the second most common cancer affecting women, you know worldwide, accounting for 10% of all cancers, it accounts for 10 % of all cancers and every woman is at risk, whatever the age, and that’s why it is very important that women in developing countries to be aware, that’s why the awareness must go round, to prevent cervical cancer, and eh, the cancer develops on the, in the cervix, that’s why they are saying cervical, or the cervix, that’s the neck of the uterus, the cervix prevent infection from getting into the uterus, and it is the cervix that the cancer affects, and eh, so many viruses can cause cervical cancer but ehm, it has been studied that we have the human papilloma virus, that causes ehm the cervical cancer the most, it is caused, necessarily caused by a virus, but the HPV is the commonest cause of cervical cancer, and eh, scientist have had over 20 years of proving that certain, the HPV is accountable for about 90% , and eh, there are more, there are 100 types of HPV, more than 100 types, and eh, the common types are 16, 14, eh 16, 18, 45, and 31, they are the commonest and these ones, cause about 80%of all cervical cancers, and eh, one can get infected through sexual intercourse, and eh, every sexually active woman carries the risk of having it, in fact about 80% of women are infected with some type of the virus, at one time in their lives

I:So ma, how can it be prevented?

R: We can do something about it, number one, we screen every woman of child bearing age, should make sure they go for screening, and eh the type of screening we are talking about is the pap smear, that’s the test they are doing in so many countries all over the world and it should be regular, and eh, it will show us and detect any signs of cervical cancer, if the test is done, and there are some changes in the cervix, it means one needs to see an oncologist, but If there are no changes in the cervix, it means you can go ahead with life, and there is the VIA too but that is not, one cannot really, really rely on the result because it is just it is just by acetic acid, if you do that and it’s positive, you still have to go back and do the pap smear, and then the test, the best test is the HPV test itself, and it is not really available in Nigeria

I: how is that done

R: how, you have to, I think my sister did it, and eh some biopsy was taken and then you have to look, use the HPV kit to look into that, it is a new thing in Nigeria, it is not as common as pap smear,

I: it is not available in this facility

R: it is, it is, it is just coming and for girls that are less than 14 and are not sexually active, they don’t need to have any pap smear done, they don’t [ so ma , what is,] but the beauty of it is that we have a vaccine to protect against this HPV because we realise that every two minutes , a woman dies, that’s it and the vaccine we have in Nigeria is the Cerverix, and as for children, young girls 9 to 14, we just have two doses at first contact, you can have the first dose and then the second dose, because it is noticed that 2 doses is already enough, for adults that are sexually active, you have to give 3 doses, at first contact, at 4 weeks then at 6 months, [ that’s for adults] that’s for adult , but for girls, 0 and 6 months

I: 0 and 6 months, for girls, then 0 ,1 6 months for adults,

R: yes, and the 1 stand for the 4 weeks , alright, because it is noticed that if you give it twice, there is more compliance, it is easy to give then its more affordable and one less injection, and it is 99% effective, but if you can’t come at the right time, it is flexible,

I: okay, so it does not have to be 0 and 6 months,

R: it should be 0 and 6 months, but it can still have it, like I miss it, now does not mean, I cannot continue with the dose, [okay,] I come first time and I am supposed to come back since 6 months at least I can still have it between 5 and 7 months,

I: so it is just a month after,

R: yes, but if you don’t wait till six months, and you taken the second dose, it means you have to take more than 2 doses,

I: If you don’t wait till six months but the person

R: if you get the second dose before six months then you need to get the third dose

I: at that six months, [ yes] after, why is it that was

R: because, it is given at six months, and you are in a hurry, you can’t wait for six months, so, if you give the dose before 5 months, so the third dose must be given, how do I say it now, for effectiveness, but if you wait for six months, then it means you will be alright

I: ma, have you had any training on cervical cancer, have you heard any training

R: we have had several summits, organised by the GSK, and

I: that’s the manufacturer of the vaccine

R:and even TOT thereafter to step down the information to ourselves,

I: what do you think will be the benefit, if this vaccine is introduced into the routine immunization schedule

R: the benefit, at least, it will lower the risk of cervical cancer to may be let me say 90%, you know the ladies are our future and then we can start now to protect them, it means cervical cancer can be eradicated and since it is very well tolerated, there has been no adverse effect even though we still have injection reaction, the redness that sometimes happen, but nothing more than that, those who use it have had sustained immune response, we know about gardacil , we have heard about gardacil but it is not available,

I; so that’s another type of vaccine

R: yes, HPV vaccine,

I: Gardacil, ma, what do you think will be the concern, the disadvantages, if this vaccine is introduced, what will be the disadvantages if the vaccine is introduced, do you have any concerns

R: what I can say is the concern. I don’t think there is no disadvantage, but the only concern is if the person has cervical cancer that is not of HPV, and has had Cerverix vaccine, you know we said it is the most common cause, there are other causes, other viruses, per chance, the person may have cervical cancer from other causes and come up with cervical cancer but asides that, nothing, there is no disadvantages, all I am seeing is advantages

I: so it has more benefit than disadvantages, but if it happens that someone comes down with cervical cancer that is not of human papilloma virus

R: it means you have to investigate and with investigation, you will know what it is, if you screen for HPV which we know is the commonest cause of cervical cancer, and it is not, the cervix is normal,

I: thank you very much ma, ma, do you envisage any challenges, if this vaccine is made routine, may be something that is related to the vaccine itself, or to humans, what do you think may be the challenge

R: it is not affordable for everybody, because it is expensive, it is one of the most expensive vaccines we stock, you see, and if you are not determined and you don’t see health as an asset, you may not take it as priority, so you have to be

I: when you say that, how much are we looking at

R: we are looking at 8000, for a dose in UCH now, In Lagos 15000, but we do ours for 8000, it used to be 6000, now the manufacturer is telling us that things are expensive, the dollar is going up and all that, so if you want to get it in UCH, it’s 8000 per dose, and look at 8000 at this time, we call recession, so people will say let me just use this 8000, so if you really know what it means that this 8000 will help to prevent family to prevent stress, because whenever a person is affected with cancer, it affects every member of the family, a lot of money, millions will be spent, 8000 is nothing when you compare with the care and the time spent on cancer, it will work for the pay, it will make everybody in the family have peace, so you can’t compare it, so if people can come to the realization of health as an asset, I think everybody will want to have the vaccine, all we have to do now is make sure we create awareness, there should be a lot of jingles about cervical cancer, talk about it at the mosque, in the churches, in the market places , where we find ourselves, we should go to schools and talk about it at PTA, invite them, go to several schools, their PTA or parent consultative forum and things like that and speak to mothers, and parents about cervical cancer, so if we do that and some people have been showing interest, and you know, as women, women are all over, they multitask, women are indispensable and if the woman breaks down, it means the whole family has broken down, so you must make sure we take care of our girl and women of child bearing age too, take care of ourselves,

I: are you saying that awareness is very low [yes] apart from finance and low awareness, is there any other thing as a challenge, do you think introducing the vaccine will bring with it

R: may be ethnicity, culture, myths, and superstition but the moment you get at them, you can’t force anybody but you can encourage, a lot of people

I: okay ma, I want to ask, you said you have been to school, parents, and others during PTA meeting, how will you say they accepted

R: well accepted but when you now talk about the price, you now hear some murmur, but when you talk generally, some people will give instances of what those who had the cervical cancer, that they had better get the 24,000, than spending millions on radiotherapy, chemotherapy and eventually death, so we still need to understand so many things, but the thing is, so many people don’t take health as a priority in Nigeria, they say that our fathers, mothers don’t have anything like that but they did not think that as things like that comes up, may be our diet is now faulty, our way of life is now faulty, then it was not faulty, it is faulty and there is a remedy, because we can’t live this lives again and it is better we go for the remedy, people are talking about cancer of the cervix, and there is a remedy, it is the only cancer that can be prevented, and with the vaccine, what stops us, even malaria, they are still working on the vaccine and in developed countries, it kills, but because of our own kind of nature , we can still manage it, in those days, children die of tb, measles, diphtheria, whooping cough, is introduction of immunization against these diseases, we don’t hear all this things, pertussis no, it not common, measles even if it happens is not common in children, poliomyelitis, and it is because of the vaccine, so, it’s a good thing we have something to protect against cervical cancer

I: ma, do you have anything to add to all we have said, may be an addition, a recommendation,

R; the government can include it in the Nigeria immunization schedule, It is a good thing, it means people will be protected and those who are protected will have effect on the country as a whole, it will be more beneficial if it is well subsided, you see once it is free, you see a lot of people rushing into it , we talk to the government so that we will really see some things as priority, we will always be very productive , economically,

I: thank you very much for your time ma, that will be all ma
