# Supplementary material for: Facilitators and barriers of healthcare workers’ recommendation of HPV vaccine for adolescents in Nigeria: views through the lens of theoretical domains framework
Source: BMC Health Serv Res. 2022 Jun 25;22:824. doi: 10.1186/s12913-022-08224-7 (PMC9233785; doi:10.1186/s12913-022-08224-7)
Supplement: Supplementary file 3 — Additional file 3. [file 12913_2022_8224_MOESM3_ESM.docx]

**INTERVIEW ID: 170210_001**

**TYPE OF INTERVIEW: IDI**

**PARTICIPANT: NURSING OFFICER, COMPREHENSIVE HEALTH CENTRE, KOLA DAISI FOUNDATION, IBADAN**

**NUMBER OF PARTICIPANT: 1**

**INTERVIEWER: T**

**TIME OF INTERVIEW: 19:39**

**LANGUAGE OF INTERVIEW: ENGLISH**

**VENUE OF INTERVIEW: KDF HEALTH CENTRE, YEMETU, IBADAN**

**AGE OF PARTICPANT: 36**

**GENDER: FEMALE**

**DATE OF INTERVIEW: 10-02-2017**

I: good afternoon ma, I am XXXXX, I am here to explore your knowledge and understanding of cervical cancer, Human papilloma Virus and Human Papilloma Virus Vaccine and I want to ask if I can record this conversation

R: it’s okay

I: thank you very much ma, before we continue , I will like to know a little about you without mentioning your name, you can just tell me, how long you have been working,

R: I have been working like 6 years now at UCH, I am 36 years old

I: what is your designation

R: I am nursing officer 1 and I work in Kola Daisi foundation centre

I: ma, can you tell me what you know about cervical cancer, have you ever heard the word cervical cancer

R: cervical cancer, just like I said is simple an abnormal growth affecting the cervix, it can be benign or malignant

I: how did you get the information you have about cervical cancer

R: we were taught about it, and I read about it too in school

I: you were taught in school and you also read about it, at what point in school

R: in my second year, or third year there about and I also read on my own too

I: was it online or in books

R: in books and when we were in school, we did somethings about cervical cancer too

I: okay, what do you know about the prevention, can you explain what you know about cervical cancer prevention, how can it be prevented

R: yes, I am aware of cervical cancer screening, what is it called now, pap smear, I know that that is one of the way to know if one has it so that one can be treated in time, prevention, well, knowing it on time could help to take some necessary steps, and then the vaccination stuffs now that we have been hearing about can actually be a good way to prevent getting it, if one has not gotten it, and then, I mean if one has not been infected, knowing, how to know is by doing the test, by doing pap smear that is the way to know but for those who don’t have it, The vaccine will be a good way of preventing it, like another thing we were told that, it is present in the semen, I mean the virus that is responsible for cervical cancer, abstinence and all of those things, keeping yourself faithful to a partner, may be condom use and one of those things too could actually be a way of preventing cervical cancer too, avoiding the virus causing it, whichever way, whichever form

I: so there is a virus causing it, can you tell me about the virus

R: the virus causing it is human papilloma virus and it is present in the semen, of course it is through sexual intercourse that one could get the virus,

I: thank you very much ma, have you had any training apart from the school own, you mentioned that you had to go online, and you read it school, have you had any training asides that

R: no, I have not.

I: you have not had any training on cervical cancer in the course of your duty

R: I have not, I cannot remember any training, I have not done any training on cervical cancer, what you are asking me, is it not about the special training in that aspect

I: training, yes, any information, may be a seminar or something

R: I can’t remember anything as such

I: okay, the knowledge you got from school, at what level were you taught

R: in school of nursing we were taught about cervical cancer, when I did my B.NSc, we did that too

I: at what level

R: I think that was in maternal and child health, that should be like 400 level

I: 400 level, so you got to know about it in 400 level [yes] you told me that human papilloma virus is in the semen and the virus is responsible for cervical cancer, is there any other thing you will like to add to that

R: how do you mean

I: as in what you know about human papilloma virus

R: I can’t remember anything right now,

I: okay, may be you will remember as you move on {respondent laughs}, let’s move on to the vaccine {laugh continues}

R: it is okay

I: what do you know about the vaccine

R: well not much, what I have heard about it is that, the vaccine will help to prevent cervical cancer, okay then, I think they said the vaccine can only be given to those that are not exposed to sexual intercourse and all of that, bear children to prevent cervical cancer

I: do you know the specific name of the vaccine

R: I don’t know the name of the vaccine

I: do you know anything about the doses, the schedule and all that

R: I can’t remember, I can’t remember, I can’t remember

I: so what does the vaccine do

R: the vaccine helps to prevent against cervical cancer

I: okay, do you know anything about the recommendations for the vaccine in Nigeria

R: sorry, I don’t understand the question

I: okay, who are those that can take the vaccine, who are those eligible for the vaccine

R: I really cannot say this is the age range, but I know children and those that have not been exposed to sexual intercourse that can take the vaccine, so that could also take the vaccine and all that, so long as one has not been exposed

I: can you tell us about the, what do you think will be the benefit, if the vaccine is introduced into the routine immunization schedule,

R: it will be a good thing, during which, because with the rate at which cancer is spreading, it is alarming actually, it will help to reduce the mortality rate, people will not be dying of cancer like before again, it will curb a lot of things financially , economically, it will go a long way, bring rest to people because if one has cancer, one will spend a lot too, and in this days of change and all that, so many people will die not because , well, so many people will die early , because they don’t have what it takes to care for themselves because that is why the vaccine will go a long way in preventing many things,

I: do you have any concerns or maybe fear, disadvantages, or may be something that may not really be of help, like the challenges that may come with introducing the vaccine

R: disadvantages

I: or concerns, things that really should be looked into before the vaccine is made routine

R: well for me, when I heard that the vaccine is only meant for people you know of a particular age or people of certain conditions and all that, for me, I feel something should still be done even to those that have been sexually exposed, if they don’t have it yet, something should still be done about them, maybe now it covers for everyone, I don’t know, I am not sure of that, if it is still for those who have not been sexually exposed, then what happens to the people that have been sexually exposed, is it that those ones are not eh, are not supposed to prevent against cancer of the cervix, that is one, I don’t know. Then, the cost, if the cost, the price is much like we have some special vaccines now for children, that are quite expensive, if this is also like that, if it is one of those vaccines that is expensive ,it will not be easy for people to get it, that is the truth, but I wouldn’t know if it is free, is it free?

I: no, it is about 7000 per dose

R: 7000 per dose, and I don’t know for how many doses

I: you take 2 doses

R: 14,000, that will not be easy for people, it will not be convenient for people , that is the truth, most people will not be able to afford that, vaccines that are not up to that, people cannot afford them not to now talk of vaccines of 7000, the cost implication is a concern, the set of people that will take it is also a concern, then the availability, because we have seen some vaccines that , okay, when you go to the clinic you want to get some vaccines for your children, they tell you that particular vaccine is not available and sometimes you are even referred to another place to get it, a thing like that will not be convenient for mothers that is the truth, for individuals who want to get the vaccine, it will not be convenient for them, if it is not readily available, [availability too ,is] availability is also a concern, ability to maintain or to sustain what you have started, not just starting it and stopping halfway, continuity is also a concern

I: Thank you ma, do you think there are any challenges that may come in should the vaccine be introduced into the routine immunization schedule

R: I think the cost price may be the issue, majorly, people will love to have it, but they might not be able to afford it, that’s the truth,

I: is there any reason why you will not recommend the vaccine

R: for people, [yes ]no there is no reason, this is about life, there is no way people will not be interested, we have seen patients with cervical cancer, people with different and all sort of cancers, at the end of the day, the end result is death, there is no reason why I will not want to recommend a thing like this, if vaccines like this, could be provided for other aspects, it will also be a good thing not for cervix alone, it is not only the cervix that is affected by cancer, there are other places, the breast, people have different kind of cancers, at the end of the day, they just go away like that, they die, if vaccines could be provided for other aspects, it will be a good thing, that’s what I think

I: Okay ma, have you had to recommend the vaccine?

R: no

I: why

R: [ hesitates, almost stammering] number one, I really don’t think UCH is into it fully[okay] I am not sure, but if the vaccine is available, why won’t I, is it available in UCH

I: in ICH

R: ICH

I: institute of Child Health, it is available, but in UCH, that’s GOP, I am not sure

R: if it is available, why not, just that we are not so sure if it UCH is fully into it, well awareness about that has been on and all that, I am not sure UCH is

I: do you think the awareness is enough considering that even you has a health worker , you don’t have information on the schedule

R: the awareness is on, is just that , it is not full, you don’t get full information, for you to say you are fully aware, that means you have information on so many details about a thing, but somehow, you get to pick somethings, here and there, even though you don’t have full information on it, you get to pick things here and there, when you go to clinic, they get to , but a situation whereby you still have your own issues, most times people don’t get full information of what whoever is giving the health talk is saying because they have their own personal issues too, sometimes we get to hear about it, you have posters about it, just that the full information is not there, I don’t know, maybe because I have not been opportuned

I: you have seen cases presented

R: yes I have

I: do you know if there are specific names, may be names they have in the localty [ you mean from the community], is there a way they define this thing

R: well , of course they would have done some investigations before they will be able to come to the conclusion that this is what the person is having but somehow, some of them will present with certain things like they have been spotting, saw small small dots , pains, in the pants, some of them will say, when they are having sex , they have pain and which has not been like that before, you understand, based on what the patient tells you, that will determine how to or to suspect what the patient may be having. Some of them will say that they may continue to spot for a long time before they now stop again, they are having pain when they are having sex with their husbands, you know they have discomfort in the private area they won’t be able to explain, they just know there is discomfort

I: when they present with that, is still early, can you say they have presented early,

R: hmm, I won’t, I won’t want to say that, well it depends, it depends, I think for people like that, it might not really be late for them, for people like that, it depends on what the person notices before the person will bring herself to the hospital but usually those are the things they will first notice, I think it may still be early until when tests and all that prove otherwise

I : thank you very much for your time, we really appreciate it

R: laughs
