# Supplementary material for: Facilitators and barriers of healthcare workers’ recommendation of HPV vaccine for adolescents in Nigeria: views through the lens of theoretical domains framework
Source: BMC Health Serv Res. 2022 Jun 25;22:824. doi: 10.1186/s12913-022-08224-7 (PMC9233785; doi:10.1186/s12913-022-08224-7)
Supplement: Supplementary file 4 — Additional file 4. [file 12913_2022_8224_MOESM4_ESM.docx]

**INTERVIEW ID: 170201_001**

**TYPE OF INTERVIEW: IDI**

**PARTICIPANT: NURSING OFFICER, IMMUNIZATION UNIT, STATE HOSPITAL, ADEOYO, YEMETU, IBADAN**

**NUMBER OF PARTICIPANT: 1**

**INTERVIEWER: T**

**NOTE TAKER: YYYYYYY**

**TIME OF INTERVIEW: 24:47**

**LANGUAGE OF INTERVIEW: ENGLISH**

**VENUE OF INTERVIEW: YEMETU,IBADAN**

**AGE OF PARTICPANT: UNKNOWN**

**GENDER: FEMALE**

**DATE OF INTERVIEW: 01-02-2017**

I: Good afternoon, My name is XXXXX , and my partner here is YYYYYYY, we are from the institute of child health, we are here to explore your understanding of cervical cancer, human papilloma virus and human papilloma virus vaccine, we want you to express your view as freely as possible , this information you are giving to us is strictly confidential and it is solely for the purpose of this research, everything you are going to say here, there is no body that will use it against you in any way, before I continue ma , can you tell me about yourself, without mentioning your name

R: hmm, I work, I am a nurse, a staff nurse midwife, and I have, the uniform is a person that has public health nursing among other things anyway and I work here in the immunization unit at adeoyo maternity hospital,

I: okay ma, what is your age range

R: 45-50

I: thank you very much ma, can you tell me what you know about cervical cancer

R: cervical cancer as it names implies is a cancer that is a tumour

I: In Yoruba how will you describe it

R: *arun kokoro jejere* to n je *ile omo, enu ona ile omo, ile omo* is the womb, so *enu ona ile omo* is the cervix

I: where and how did you get your information about cervical cancer

R: that was from the school, the first encounter I heard with it was from the school, then later, the hospital setting, later around, you see it around, you hear people talking about it, *o ni kokoro, kini mo pe yen, o ni kokoro* cancer, *o ni arun* cancer, because before the thing is not so, it is not as many as this,

I: okay, can you tell me what you know about cervical cancer prevention, how can it be prevented

R: as I said initially, cancer is a kind of tumour, and they said the cause is unknown, as I said, I believe it’s cancer, it is a tumour that has no cause, so that answers one of your questions, that the cause is unknown, you can say this is what can cause it but as times goes on, you find out that based on research, you have people that will tell us, if you eat this certain kind of food, like smoked fish, like the rest, it can prone people to cancer, then there are somethings that some women are into that can prone them into somethings, like cancer, like certain drugs, introduction of certain things into the private part especially into the cervix, douching, I heard it too, I don’t know how far that one is true , I don’t know how true is it

I: so how can it be prevented

R: how can it be prevented, the first thing I know is that, you make screening, this cervical screening, even before getting to that, adolescents, I think from age 10, a child that is not yet exposed to sex can take Cerverix, that is the vaccine that prevent the occurrence of cervical cancer and the person that is exposed has to go for cervical cancer screening before getting the Cerverix then as I said there are some drugs that are introduced into the body that can make a person prone to cervical cancer then the person can also have it, I don’t know if you have heard about this things, that somebody can get it from generations

I: like its genetic

R: I don’t know, I don’t know, I don’t know how far that one is true, I can’t discuss that, then this Yoruba people believe they can affect a person with cancer,

I: spiritually

R: yes, spiritually, then, the prevention, I said introduction of certain things, so we should health educate people , the women especially not to be introducing a lot of things on the premise of saying they want to have an abortion, do this and that into their private part, then uncleanliness, if there is an infection, I believe that as time goes on the infection will be coming and if there is no good treatment in the appropriate place, I believe as times goes on, it will turn into another thing, even if it is not cancer, it will become something else

I: you mentioned the other time that your first contact with cervical cancer was at school, can you describe the content of the training

R: we were given the definition, the causes, and the prevention,

I: so at what stage was that training given to you

R: may be second year or third year, I don’t know

I: of, is it the school of nursing

R: school of nursing

I: after you finished, were you exposed to any training on cervical cancer apart from the school

R: no I was not exposed to any training but I have seen a lot of cases like that where I work in gynae ward

I: cases of cervical cancer

R: yes, when I worked in gynae ward, those that are insolven, affected aged

I: they are what,

R: aged

I: affected aged, old people, like how old are we talking about

R: those that I know, there is a mama, she was like, if I am not mistaken the woman was above 70 years, when she had it, when she was brought to the hospital that she had it, and the consultant that was called, that was when I first knew that it is called *kokoro jowojowo*, the consultant approached the mama, and told the mama, *mama se emo pe arun ti e ni yi, arun jowojowo ni*, I was pissed , I was furious, look at this man, so careless,

I: *arun jowojowo*

R: what do they mean by *arun jowojowo*

I: that it is expensive

R: he just went straight to the woman, mama, *awon omo ti e bi, ibo ni won ti n sise*, I didn’t like the approach anyway

I: was that the only thing described as *jowojowo* that time

R: yes, yes, because it is expensive, they told us among other things, they taught us the treatment for it, you know they will expose them to radiotherapy, radiotherapy, and he told mama, mama this disease, the drugs are very, very expensive and the treatment so far is expensive, you know it has not been rampant then, what I am talking about is like 1992,93

I: that was a long time ago,

R: not very long, that was almost 25 years ago

I: not too long, how old am I, very young, that was a long time, then It was described as *arun jowo jowo*, what can that name mean, was there any other name given to it apart from *arun jowo jowo*

R: like I said spiritually, people will call it *ota*, *won so n iota ni*

I: so *won so eni yen ni ota loju ara*

R: I don’t know{laughs}, like I know of a person that has this cervical cancer, *ah o ma se, awon omo ma* suffer, *obirin , bi oko se ku ni yen, won so lota ni,* that is what people perceive , *nkan ti awon eniyan se* , it is spiritual , that people they are after their wealth, they are after this or that, so one way or the other they eliminated the husband

I: okay ma, can you explain human papilloma virus

R: it is a disease caused by virus as the name implies, and anything viral infection, we are taught that it does not have a cure but if it were to be some other ones like these chicken pox, usually the thing will go, but in the case of cancer , even after preventing it, there is a woman that has it, she had it really because she died, she just died last year, like they told us, it does not have any cure, the only thing is they can give them treatment, I believe that if it is abroad, they will tell the person the amount of year, day that the person will spend in life, because I have heard of a case like that, they had to record the woman’s activity before the last days, the last days really so that is it, it is a viral infection like I said, it will be very painful, { the virus itself} initially it may not be painful but just to take this pain relievers that we take

I: thank you very much ma, what do you know about human papilloma virus vaccine

R: it is the vaccine that is used to prevent the occurrence of cervical cancer

I : do you know the types

R: like the one I said, that of Cerverix { apart from Cerverix} two doses for the younger ones, three doses for the older ones

I: younger ones, what age range is younger ones

R: I think 9,10 up to 16 I think, I am not too sure but anything above 16, 3 doses

I: but for those that are between 10 and 16, two doses

R: 16 and even 9, I think it starts from 9

I : so ma, what do you think is the importance of the vaccine

R: prevent the occurrence

I: do you think it is important { hmm} how? Why?

R: to prevent cancer, It is important, the case is now rampant, the occurrence is, the record is much than before

I: what is the recommendation for the vaccine in Nigeria

R: do we produce it in Nigeria

I: most of the vaccine we use are they produced in Nigeria?

R: no,

I: is the same thing, what is the recommendation for the vaccine use in Nigeria

R: I will recommend it strongly

I: is the vaccine available

R: yes of course

I: do you give the vaccine here

R: no, we don’t, but we know about it, I can give my children , we can give our children, those that are up to the age give them and if they come to you, you direct them to where they can get the vaccine, if you want to educate others but you can refer them to where they will be given

I: what do you think will be the benefit of introducing the vaccine into the routine vaccine schedule in Nigeria

R: it will reduce the occurrence of cervical cancer and it will caution, if they know that they have taken certain vaccine to prevent this, you know people will now be aware of this cervical cancer and how to prevent and I believe in a way, it will caution some ladies from being promiscuous

I: how will that happen

R: you know among the health education they will be giving them, it will not be one way, it will be diversified, you taught them the personal hygiene, you teach them good food, you know that good food is part of the cause, if the person is not eating well, you tell them the way of life to live, how to live well as a woman and then they will now tell them the vaccine is okay for you ,the person that is not exposed, you take this, the person that is thinking of going into such things, will think about it, when we were in school, I heard someone telling me, we want to know if we are mature or not, we will have to be exposed a little bit, a person like that will be cautioned, so I believe one way or the other, it will have aN impact on promiscuity

I: so if you are not exposed, you won’t want to be exposed

R: I believe so, because hearing such a thing, even if it will be, you health educate them, trainings for them, along that, you tell them whoever is interested in having the vaccine, you have this introduction

I: so ma, what do you think will be the disadvantage

R: how can there be a disadvantage to what is preventing what is not good

I: so there is no disadvantage

R: I don’t think there is any disadvantage

I: nothing like something of concern, or fears

R: it is lovely like the routine vaccine you give to children, no concern, no fear, like this polio vaccine, we know that it has reduced, apart from, according to WHO or whatever in Nigeria, they said up to zero level, that is polio, polio like 20, 30 years ago, polio is rampant, even this tuberculosis, the BCG that they give the children, so this ones too, they equally bring goodies, no disadvantage, no disadvantage

I: do you think there are any challenges that may come up if the vaccine is made routine

R: of course, financial constraint is number 1, you know we have that challenge, that’s number one , that is affordability, but now I said , here, people have been giving it , some places have been giving it, we have not been giving it here, they have introduced it to us, we know about it, we know what may come up, if we give it, but we have not been given the go ahead administratively

I: administratively

R: there are some people that does not believe in giving this vaccines, they say that what they want to give is taboo, that they want to reduce the population of people in Nigeria, that one can affect, the may be ignorance, so people will not want to take, if they start giving women now, they said that they want us women to be giving birth to one child, so that is ignorance, lack of adequate knowledge, then time factor, someone that does not know about the vaccine, you now tell the person come tomorrow, come and take this vaccine, the person will tell you that the money that I will spend to take the vaccine, I will use it to feed my children, by the time I use the money to pick one or two things in the market, I can’t waste my time, so time factor, so many things anyway, then scarcity, the scarcity of the vaccine, it is not something that you will see in your health centre, that will be readily available, I don’t think so, if the vaccine is sometimes not available, it may not be accessible

I: is there a reason why you will not freely recommend this vaccine

R: there is no reason

I: you don’t have any reason

R: I don’t have any reason, if the person is not exposed

I: if the person is exposed what is the way forward

R: do cervical screening

I: so ma, the last question I want to ask, can you give instances when you have recommended this vaccine to adolescents

R: yes, my friends, friends that have ladies, girls, I advise them, I advise my clients ,you know we have a lot of mothers here, I tell them about health education, I tell them we still have a lot of vaccines for your older children especially girls, I tell them, I tell them, one on one, one on one, when we are discussing things, some women that have so many children, like seven like eight, I tell them there are lot of things they can take, how old is your first child, how old is your daughter , how old is she, they will be asking about HPV for their child , this and that

I: Ma, before we round up this session finally, how do you think we can overcome some of this challenges you mentioned , financial constraints, ignorance, time factor, scarcity of vaccine, how do you think we can overcome?

R: yes, the first thing is for the government to be ready, if the government are ready, you know it is one thing for this, you know the government has a plan for taking care of age zero to five and pregnant women, they should take into consideration too, the adolescents, so they should include this vaccines into the routine immunization that we have, they should introduce it , they should make it free, you know all the vaccines that we give here are free, we don’t give it here because it is very expensive, initially there is one that we don’t give here too , PCV and at some other places they give them 6500, 7000 and when they made it free, we give them free as well, so if the government should make it free, that is a step ahead, [okay] if they make it free, it is a step ahead, then propaganda, you know the, go to media, they will advertise it, *iya lagbaja*, and it will be in a language they understand, you come down to their level ,it will be a language, they come down to their level so that those people will be able to understand, it is not a grammar that they will be speaking, come down to their level, so that they will be able to understand, especially the market women, *eh, kilon sisie fun, won tan yin , kini kan kini kan*, they will disabuse this taboos, that they will be carrying about, turning this information upside down, so they will tell them, they should, anyway they know how to do it , I don’t need to emphasize on that, if they want to make it affordable, they know how to go about it, that’s the first step. The government should make it affordable and propagate so that people can know about it, tell them in a language they understand, how it works and what it works for, they make it easily accessible, yes, yes, go to different health centres, you are able to see it, is not that *kin to wo motor*, where do I even get transport fare, but if it is very close to you nearby in your own centre, you know we have centres here and there, then they should let the health personnel know that this is free and free, that is one of the constraints , that if you get to the centre or to the hospital, what is supposed to be free will not be free, they will say it is a token that if you go to the other side, some people pay 7000, they are just being nice, they have just reduced it to 1000 and this is something that is supposed to be free, so they should, they should look into such areas too.

I: in that instance, is that the fault of the health worker or who?

R: of course, it is the fault of the health worker, they give them free and it is supposed to be free, there are some people that are, what do I call it, they are greedy, so they will be tasking, even 500 some people cannot afford to take this vaccine, so it should be free and free

I: thank you very much for your time ma [ you are welcome] we really appreciate your decision to discuss with us, and we assure you that everything that we have said in the course of this interview is confidential
