# Supplementary material for: Facilitators and barriers of healthcare workers’ recommendation of HPV vaccine for adolescents in Nigeria: views through the lens of theoretical domains framework
Source: BMC Health Serv Res. 2022 Jun 25;22:824. doi: 10.1186/s12913-022-08224-7 (PMC9233785; doi:10.1186/s12913-022-08224-7)
Supplement: Supplementary file 5 — Additional file 5. [file 12913_2022_8224_MOESM5_ESM.docx]

Topic: Knowledge of Cervical cancer, Human Papilloma virus and HPV vaccine

Moderator: A

Note-taker: DDD

Identifier code:

Language: English

Number or participants: 1

Date of Interview: 01/02/2017

Time of activity: 00:19:26 (hours: minutes: seconds)

Date transcription completed: 06/03/2017

Transcription completed by: A

I: good morning ma, my name is AAAAAA. I am here to explore your understanding about cervical cancer, HPV and HPV vaccine. I implore you to express yourself freely as everything you tell us will be kept confidential. Before I can go ahead with the questions I need to know that you accept to do this interview without being pushed into it. Ma, do you accept to be a part of this interview? [Yes] and you accept that I record your voice? [Yes]. Thank you ma, can you please introduce yourself?

R: I am a nurse; I am working with Adeoyo maternity teaching hospital. I am a registered nurse, midwife and public health nurse. So I joined Adeoyo in- I have been in service since 2000 but I joined adeoyo in 2014. I am a Nursing officer two.

I: how old are you ma?

R: I am 44years old

I: ma, can you tell me what you know about cervical cancer?

R: cervical cancer is a cancer that affects the cervix and it mainly affects ladies and it can affect any lady. Someone that has already reached maturity, maybe the person has been exposed to sex or the person is still a virgin. It can affect any lady especially the woman because it concerns the cervix.

I: can you explain what you know about the prevention of cervical cancer?

R: the person can go for cervical examination, I think they said they are doing it at uch or other health facilities, or you can take this vaccine to prevent cancer.

I: so asides the vaccine, are there other maybe methods to prevent cancer, are there home remedies that people can use?

R: they said we should avoid cervical douching and then we should avoid washing our private part with soap or any insecticide or anything that can kill the normal flora of the body that can also cause cancer for the cervix. [Okay] then when we want to wash our private part, we should use only water to prevent it. And I think one should avoid multiple sexual partner because that can also cause it and…. And so on

I: during the course of your training as a nurse, were you taught about cervical cancer?

R: yes

I: what were you taught?

R: they taught us what can cause cervical cancer, how to prevent it, the vaccine you can use to prevent this cancer and the age range that can be vaccinated with this vaccine

I: and what is that age range?

R: I think they said it is 11years to…. 45years or so [that can contact the-] that can get the vaccine

I: what of the cancer itself, what was the content of the course? So they told you the prevention, what exactly did they teach you about the cancer?

R: they said the cause of the cancer is unknown but there are some predisposing factors that can cause this cancer that I have mentioned earlier such as multiple sexual intercourse, through douching or using antiseptic soap to wash the vagina or maybe you come in contact with someone that has the cancer or you are sharing toilet with the person or you are sharing undies with someone that has the cancer. These things can predispose someone to cervical cancer.

I: at what stage of your training were you taught all these?

R: they taught us during my midwifery course

I: what is the duration of the midwifery course?

R: it is one year but then it was 18months

I: is it just a level or there are different levels?

R: the topic is part of midwifery course

I: so it is a topic on its own? [Yes] so moving on, what do you know about human papilloma virus?

R: I think…they said it is a vaccine that they give to women to prevent cancer of the cervix. And then I went to this seminar that GSK gave at uch, they told us the age range but I have forgotten, the age range they can give the vaccine to prevent cancer. I think its 2 or 3doses, I have forgotten

I: okay that is the vaccine but the virus itself …human papilloma virus; do you know anything about that virus?

R: I don’t know much about it…I don’t know anything about the virus

I: okay. In this community, have there been cases of cervical cancer that you know of or that you have seen?

R: in this facility?

I: not necessarily in this facility, maybe during your training

R: no I have never seen a case of cervical cancer

I: not even outside the hospital?

R: no

I: because I want to ask that do you think people in the community know about cervical cancer?

R: I think so but maybe they could have heard through the media, but I don’t think they know much

I: are there some names they call cervical cancer, asides the English name that we call it?

R: I don’t know

I: okay ma. Moving on you said you don’t know what HPV is. So the HPV is the organism causing the cervical cancer so it is the virus that is transmitted during sexual intercourse and can now lead to cervical cancer. [Okay] and to the vaccine, you said you do not know the age range that they give

R: yes, I said I have forgotten

I: okay, are there different types of the vaccine or is it only one

R: …I think it is one that I know

I: which one is that?

R: the one the GSK people brought. I know the GSK Company produce one but I can remember the name. They said they have a vaccine that prevents cancer

I: do you give the vaccine here?

R: no. in this unit? [Maybe in this facility] maybe they give it at uch

I: do you know the schedule?

R: I don’t know

I: what is the importance of the vaccine?

R: it is just for prevention, to prevent people from getting the cancer

I: any other thing asides that? Any other importance that you think can be associated with the vaccine?

R: ….I think it is just to prevent cancer

I: do you have recommendations towards making sure people use the vaccine or maybe in the production of the vaccine?

R: I have not seen the vaccine, I only heard about it but at times there are people that come here to ask for the vaccine but we refer them to uch because we don’t give it here. We tell them to go to uch so they can get the vaccine but here we don’t give it

I: but you have had cases of people coming here to ask for it?

R: yes but we normally refer them to uch because we do not offer that service

I: do you know why adeoyo does not offer that service

R: maybe because it is costly or maybe…you know in adeoyo most of the people that patronize us are illiterates, they will be asking for free things, maybe that is why government did not bring it here. But at uch, they know that nothing goes for free

I: you mentioned that it is costly; do you have an idea of the cost?

R: I don’t have an idea; I just know it is costly. I know it is GSK that produce it, I don’t know if there is any other company producing it but I know of GSK and I know their things are costly [okay] but I don’t know the price

I: what do you think about introducing the vaccine into the routine schedule in Nigeria?

R: it will be okay and people will appreciate it but there are some people that want to get the vaccine but cannot get it because of financial constraint. But if they introduce it to the normal routine people will have access to this vaccine and it will even reduce cancer of the cervix in the society

I: so you have mentioned the benefits, do you think there are some disadvantages of introducing the vaccine into the routine schedule?

R: there is no disadvantage except if people abuse it, maybe they will not give it to the right people. They may be hoarding it and selling it outside, asides that it is very okay

I: can you foresee challenges that may arise as a result of introducing this vaccine into the routine schedule?

R: I think the only problem that can arise is in storage, I think it is supposed to be in a refrigerator but due to constant power failure, the potency may be reduced when there is no storage facility, I mean in state hospitals. Asides that, there are no other challenges

I: okay, from what I know, it has been said that people should get the vaccine before sexual initiation which means we should be targeting our adolescents, do you think it is okay to give people of that age considering you said that one gets the cancer from having multiple sexual partners. Do you foresee any challenge in introducing it to adolescents?

R: there is no challenge there. These days a 9year old girl is already exposed to sex and we usually hear form the media. Even 7year old girls should be allowed to take the vaccine because they are already having sex. They are exposed so maybe they should even add it to this normal immunization routine they give to babies like this BCG, they should add it to it. so they can be immunized against this thing while they are babies before they get exposed to it in the future

I: is there any reason why you will not freely recommend it to an adolescent?

R: there is no reason. Adolescents can have it so far they are prone to sex especially the ladies

I: and you don’t think- someone once mentioned that she will not allow her adolescent to take it because the adolescent will feel that she can have sex anyhow

R: you will just have to health educate them that this vaccine that we are giving you does not protect you from other sexually transmitted diseases, it will only prevent you having cancer and the cancer is not one of the STDs. We will let them know someone that gets the vaccine can still have HIV, so we will health educate them, make them know the importance of this thing and how it works for them. That this one does not give free access to have sex around

I: are there reasons you can think of that may not make an adolescent take the vaccine?

R: they may be scared that this thing can affect them in the future, that maybe they may not get pregnant in that future, that it can affect them. All these taboos can affect them but we will let them know that it cannot affect them, that it does them more good than harm. We will talk to them and enlighten them on the importance of this vaccine and some of them will turn up. But despite that, some will still say they do not want it but we will give them health talk on it

I: apart from the fear of side effects, are there other things that you think- let us even use this community as an example, are there reasons why you think adolescents in this area may not go for the vaccine?

R: …. …due to the level of their education, this place is very rural. Due to their level of education, some of them will say they do not want it, they can you say you want to kill them that this is the way the government wants to reduce the population of Nigeria, they will think of different things. But we will enlighten them that this vaccine cannot affect them, that it is just to prevent them from having this cervical cancer in the future.

I: you as an individual, will you allow your adolescents to take the vaccine?

R: I will allow them

I: can you describe an instance where you have recommended the vaccine to a patient?

R: I have never recommended the vaccine to any patient. I know of it but I have never had to recommend it to any patient.

I; do you have any other comment you want to add that may help this research?

R: my comment is that maybe the government can just introduce it to- as you said earlier they should add it to the normal routine of immunization so that people can have access to it and it should be free because all these vaccines we have here are free. If it is free, people will have access to it and they will get it. As this vaccine- pcv is- pneumococcal vaccine was 6500 in uch and people said they cannot pay but now WHO has added it to their normal routine and we are giving them free here. Now people come for it so if this vaccine is also free, people will come for it and even with our own effort to health educate them, I know they will turn up. And then we can reduce this cervical cancer in Nigeria

I: thank you so much for your time ma; I really appreciate you talking to us despite the fact that you are not feeling too good. Thank you so much ma

R: you are welcome
