# Supplementary material for: Facilitators and barriers of healthcare workers’ recommendation of HPV vaccine for adolescents in Nigeria: views through the lens of theoretical domains framework
Source: BMC Health Serv Res. 2022 Jun 25;22:824. doi: 10.1186/s12913-022-08224-7 (PMC9233785; doi:10.1186/s12913-022-08224-7)
Supplement: Supplementary file 6 — Additional file 6. [file 12913_2022_8224_MOESM6_ESM.docx]

Topic: Knowledge of Cervical cancer, Human Papilloma virus and HPV vaccine

Interviewer: T

Note-taker: D

Identifier code:

Language: English

Number or participants: 1

Date of Interview: 1/02/2017

Time of activity: 00:24:05 (hours: minutes: seconds)

Date transcription completed: 06/02/2017

Transcription completed by: A

I: so as I said earlier, my name is XXXXX and my partner is DDDDD. We will be asking you questions about your understanding and knowledge of cervical cancer, Human Papilloma virus and HPV vaccine. Before we continue, I will like to ask for your permission to tape the conversation, can we tape the conversation ma?

R: yes

I: Thank you very much ma. So ma, I will be asking you a few things about yourself. Like your designation, your years of experience, your age range and erhm.....just a few things about you. Please can you tell us about yourself briefly? What work do you do?

R: I am a nurse

I: okay, what post do you hold here?

R: The head office as matron

I: okay in Foko Maternity center. How long have you been working here?

R: I have been working....I think I have worked for at least 20years

I: What is your age range?

R: By April I will be 53

I: Thank you very much. So I will like to ask, have you ever heard about cervical cancer?

R: I think I have heard about cervical cancer

I: okay ma. Can you tell me what you know about cervical cancer?

R: well, just ehn ehn I have scanty knowledge about cervical cancer I just know that it is ehn...anything called cancer it is abnormal multiplication of cells [yes] of the (inaudible) columnar junction of the cervix. I hope you know there is a junction in the cervix? ((inaudible 0:02:19.9-0:0228.8)) ....how do I express it? it is abnormal cells. [Okay] I think they said one of the causative organisms is human papilloma virus. Repeated infection of human papilloma virus, and then there are some predisposing factors like....I think I heard early marriage, multiple sex partner, those are- the women that are predisposed to it [okay] I think I learnt that woman that marries early, those that have their menarche earlier, those that are with multiple sexual partners and it occurs to- it is rampant among women of ehn....which age is that...I think around childbearing age [okay] I cannot remember the age but I think the age is around....it has escaped my knowledge.

I: okay thank you very much ma. Ma can you explain what you know about the prevention? How can it be prevented?

R: Well...the first thing is to create awareness, to let people know what cervical cancer is, how it occurs, the causative organism or the predisposing factors. Because there is a saying that prevention is better and cheaper than cure. So that is one of the ways to at least- to let the people have a change of habit, those that are predisposed to it one will health educate them. then those that have ehn this human papilloma virus, we should let people know what it means and the infection they cause so that anybody that has such an infection will quickly go to the hospital for treatment before it degenerates. Then we should teach people to go for this checkup, there are some screens that women can do especially after childbearing... you know during childbearing there is opportunity to view the cervix at least during labour or postnatal but when a woman is done with childbearing there is need to go for screening so that the cervix can be viewed on a timely basis maybe yearly or twice yearly. And then the government should be....there should be agents in place that will support- you know our problem in Nigeria is poverty so as you have learning programs now, if you have agents that will do it for people, do the cervical screening you know early detection- if they detect it early, I learnt they do screening and treatment, this cryotherapy or so they can easily do it before it degenerates into the stages that will be difficult to avert so I think those are the things we can do to prevent it

I: okay ma, this information you are giving us, did you get it- can you tell us how you got the information you have?

R: I got it through a program that an NGO held [okay] at Ibadan North-west local government, the rotary club there organized it so it was one of their doctors that came there to give us a brief lecture and we were all opportune to do the VIA...visual ((inaudible)) and ascetic acid.

I: so you had a real training on-

R: not a real training, just a snappy short- not up to- before- not up to 30minutes lecture, they gave us, because they really came to do it. They came to screen women in their area there so as medical persons we also said we want to do the screening so they just gave us- just to empower us, atleast as you are asking me now I am able to say something [yes] so that is just it. They want to teach the nurses over there to carry out VIA, but not now. They said they will have sessions of training but the first one they did I was opportune to be there. So just around 30minutes, not up to an hour.

I: when was that?

R: just this last week

I: this last week? ((Laughs)) it is very timely [yes]

R: the thing is fresh in my memory

I: but before then, have you had any opportunity to be trained?

R: No but I have- I did cervical smear, they just came, they did it for us in the local government I was. It was an NGO also that organized it through the church. They just came to screen us, discussed with the women over there. Their own...it was cervical smear, you know there are different screening you can do, I learnt this VIA is the one that is easier [okay] so they did the cervical smear[pap smear] sorry the pap smear and they just left. No lecture, nothing but this other one we did last week they gave health talk to the women that are on the seat, we too as health workers they gave us our own. It was during our own that I was able to pick all these ones that I am telling you now

I: Okay so you did not have any contact with the information during school

R: ha...it’s been a long time ((laughs)) I passed out 1986 [haaa] from school of nursing and midwifery 1988 ((laughs))

I: it is well. Ma, can you explain what you know about human papilloma virus?

R: I don’t know much about Human papilloma virus, I just know it is one of the virus that cause problem in human body

I: you just know it is a virus

R: it is a virus [okay] and any virus- there is no normal flora for virus right? I don’t know much about it

I: what about the HPV vaccine? Have you heard about it?

R: I have heard about it through my daughter, her friend living in Lagos, the mother took the thing for her own daughter. So she said my own daughter should encourage me also to sponsor my own daughter so that she can be vaccinated but I am yet to do that. I think I learnt that they will receive the vaccine maybe three times or two times but I heard it is costly, the one in private hospital in Lagos. I have not been there but I heard my daughter saying it when she came back from her friend's house because she went there for holiday ((phone rings 0:11:50)) But I am yet to take it

I: so what are things she told you about the vaccine?

R: No, maybe when she goes there, they will give her full explanation. She just said mummy Dolapo said I should try it too, that the vaccine is a vaccination against cancer. That was all she said but since she has not been able to go there maybe when she gets there they will explain to her some of these things. But I am yet to-

I: But you know it is three doses

R: yes she told me they said it is three doses; It is the amount that now hindered-

I: how much is it?

R: It is costly ((emphasis)) I think it is over 100thousand

I: 100 ((exclaims)) that is a lot of money

R: it is from private, it is private hospital so you can’t hinder him or her from selling his commodity. If you like you come there, if you don’t like you will go to another one, another place you feel you can get the same service at a cheaper price, if you can get. That’s why they....you know Lagos now ((laughs)) that is Lagos for you

I: what do you think is the importance of the vaccine? Will you recommend the vaccine for Nigerians?

R: since I am yet to know the content but for me to have heard that it prevents cancer, if I see somebody that wants to take it I will recommend it. I am a nurse I recommend vaccination for little children and those childhood killer disease. So if it’s the same thing and ((inaudible)) so if I see somebody who wants to take it I won't hinder the person from taking it. So far she is able to afford it

I: like that person that told you- that your friend that [my daughter's friend] like how old is she?

R: I think she will be 20

I: okay even she is not a teenager?

R: but I think she started last year or the year before, I don’t know

I: when she was like 18? [Yes] okay. Do you think there are any benefits asides from prevention of cervical cancer; do you think there are any benefits if we introduce this vaccine into the routine vaccine schedule?

R: there will be benefits since it is against human papilloma virus. Human papilloma virus is not preventing...is not only cancer that it is causing, there are other infections of the body that it is also causing so if we introduce it, it will be okay because all those other infections that this virus use to cause will also be drastically reduced. You know we said the other time that it is cheaper when you receive immunization, it is cheaper than for you to have infection and now contact immunity after you get out of that infection because definitely it will lose your- you will spend money , you will spend time and the energy the body system will discharge. So to prevent all those risk stage if we have the vaccine, I think it is better. The vaccine is far better, not only for cancer but for all other infections because all these other infections can also kill. It’s not only cancer that...you know virus can- normally you know viral infection has no specific treatment. You know that unlike bacteria or fungi, so if there is any immunization against it, I think I prefer it than contacting the infection and now looking for which drug will work or which one will not work ((phone rings))

I: okay ma, what do you think will be the disadvantage, if we introduce it into the routine vaccination schedule?

R: I may not be able to say more about the disadvantage since I don’t know the content of the vaccine, how it will operate, the contact at which the immunization will be introduced, I don’t know all those things. So that one will not give me opportunity to talk about the disadvantages. [Okay] so I may not be able to say much

I: What do you think can be if it is eventually introduced?

R: Disadvantage on the part of the masses or on the part of the government or who?

I: you can tell us, it may be on the part of the government

R: maybe on the part of the government, the problem that normally ensues is that we will have it for some time and small time the vaccine is out of stock. That is the only problem we normally have. All these programs are good but the funding part of it, the funding aspect is the problem.

I: so funding may become a challenge?

R: yes it may become a challenge because I don’t know how much it will cost, you know this immunization we give to our children there are some times it will be out of stock, although it has taken longer time that it has happened. It used to happen. even now, it may not be all at a time, some- one or two of the vaccines may be out of stock maybe a month or two before there will be restocking. That is the only challenge. The other challenge that we may have is to get people to understand it, but if we start from the grassroots I don’t think that one will be a big challenge

I: okay why did you say that ma?

R: if you- because we are the people that are close to the- The PHC are the people that are close to the people in the grassroots, we know how to explain it to them in the language they understand. Like this pneumonia PCV, Pneumococcal conjugate vaccine, you know we just introduced it last year July. We started, we added it to the routine immunization, the moment we gathered women, we explained to them, the opinion leaders, we let them this is problem, this is the cause of the problem and this is the solution that the government has provided, they will embrace it. If you let them know but if you don’t explain to them it will be difficult for them to embrace it. Maybe you will be hearing the last discussion we had with one woman that came with IUCD, you know they use to give themselves information, they have wrong information but when they come here we disabuse the taboos and then we give them the right information. So when you take time to give them the right information and you assure them that we are the providers and we too we do it, they will accept. That woman came with the purpose of removing the IUCD, she said they said it can break inside; they will have to cut her up to remove it if it stays longer but we reorientate them to the right thing to disabuse the taboos they come with. We explain to them the- when those things work what they can cause that is normal. We let them know if there are disadvantages, we don’t hide it from them; we give them the advantage and the disadvantages so we will give it to them to choose. When you counsel them you will now give them the option of choosing, you don’t enforce it on them. We give them time; we allow them to choose, so that is just it.

I: so if they-

R: they introduce it and they bring it to the rural level by the time we begin to send it out- we have various kinds of meetings that we do, we have WDC meetings, we have immunization days that we gather women, we have antenatal days that we gather women, they have community meetings, landlord meetings, we go there to tell them, the opinion leaders those that are respected that are well respected in the community, the moment you tell them just take it that all the people in that community have heard and they will do it. That is the way we-

I: starting from the grassroots

R: yes that is it, from the grassroots

I: Is there any reason why you will not freely recommend the vaccine for adolescents?

R: you know I told you I do not know the content, I do not know the side effects, I don't know the cause of its action so I cannot really- since I don’t have much knowledge about it [okay ma] then if I have the knowledge now, I will know who it is for, the side effects, the mode of action and all those things, that one will make me to know who to recommend it for but I don't know it.

I: so what is your recommendation towards the success of this vaccine for adolescents?

R: it is good if it is ehn...you know like this immunizations now, not all these childhood killer diseases will ensue at the tender age like hepatitis now, I learnt the signs and symptoms will come up at around 15years and we have started the vaccine right from the day of birth, the day one. So if there is no any contraindication for adolescents to receive it, advice to receive the vaccine is good because you know the adolescents and the middle age, when they are yet to get married, all the reproductive organs are intact. I think if they introduce it at that time, I think it is okay if there are no contraindication. It is okay, it is good.

I: okay. Ma, do you know local names given to cervical cancer? Have you heard about names people use to describe cervical cancer?

R: I don’t know

I: We have heard in some instances people call it *aisan jowojowo*

R: No it is *jejere.* Cancer generally they call it *jejere*

I: okay, thank you very much for your time ma, we really appreciate you

R: you are welcomeS
