# Supplementary material for: Facilitators and barriers of healthcare workers’ recommendation of HPV vaccine for adolescents in Nigeria: views through the lens of theoretical domains framework
Source: BMC Health Serv Res. 2022 Jun 25;22:824. doi: 10.1186/s12913-022-08224-7 (PMC9233785; doi:10.1186/s12913-022-08224-7)
Supplement: Supplementary file 7 — Additional file 7. [file 12913_2022_8224_MOESM7_ESM.docx]

Topic: Knowledge of Cervical cancer, Human Papilloma virus and HPV vaccine

Moderator: A

Note-taker: T

Identifier code:

Language: English

Number or participants: 1

Date of Interview: 15/02/2017

Time of activity: 00:15:05 (hours: minutes: seconds)

Date transcription completed: 06/03/2017

Transcription completed by: A

M: good afternoon ma [good afternoon] my name is DDDDDD and with me is XXXXXXX. We will be asking you questions to explore your understanding of cervical cancer, HPV and HPV vaccine. We just want to know what you know and we implore you to tell us the truth and feel free to talk to us. We will ensure confidentiality in this and that is why we will not be using your name in the course of this recording. Do you permit me to go ahead with the questions ma? [yes I do] and I am allowed to take the recording? [yes you can] thank you ma. Ma, can you tell me what you know about cervical cancer?

R: cervical cancer like you said is a type of cancer that affects women and it affects the cervix region in a woman

M: sorry ma, I was supposed to tell you to introduce yourself, let us start from there. Tell me about yourself

R: okay, I am a senior nursing officer working in the immunization section

M: for how long have you been working there?

R: working in this area, it is been about 4years now but my nursing experience is more than 4years

M: what is your level of education, your qualification?

R: according to our own, it is HND

M: okay thank you ma. So you were telling me what you know about cervical cancer

R: I said cervical cancer as the name implies is the cancer of the cervix, it affects the cervix in women

M: have you ever seen it happen to anybody?

R: yes I have seen a lot and I have nursed some of them

M: so what was it like? How did it present? What are the symptoms?

R: most of the time or what brings them to the hospital already- that is they come in the advanced stage, when the cancer has already started because actually by the time they come in it would have gotten to the stage that they need medical help and according to knowledge it is something that has been for a long time symptomless. The actual infection has been there lying insitu with no symptoms but by the time they will come up with symptoms and signs the cancer has already started or they already have the cancer. It would have been there for a long time before they now manifest or come down with symptoms. You want to know what the manifestation is. [yes] alright like I told you, it is usually symptomless, they will not have any- they will not even know that they have problem in their cervix that it is there, until maybe later they start to have irregular bleeding. The bleeding will not conform to maybe their menstrual period. They may think they finish their menses today but in a few days they start bleeding irregularly anytime during the month they will just be bleeding. And again most of the time we call it post coital bleeding, that applies to those that are still sexually active, after having sexual intercourse they will start to bleed. [okay ma] pains, foul discharge and they begin to lose weight and become weak and tired.

M: thank you ma. Can you explain what you know about cervical cancer prevention?

R: prevention of cervical cancer? I really don’t think it is what one can prevent like that except you want to prevent it using the vaccine though there are some precautions which you can take, like the caution there is that sometimes we discourage people from having multiple sexual partner, from being too promiscuous and to be … avoid multiple partner most of the time. That is the precaution and you go for the vaccine and you have to go for regular medical checkup because there is a test you can be doing at regular interval to help you- for early detection of the problem.

I: thank you so much ma. all these that you have told us now, where did you get the information from?

R: well I read about it, I read, and it is expected of me to know some things according to my profession.

I: were you taught during your period of training?

R: yes I was taught

I: what exactly were you taught then? The course, was it just the causes, the prevention? And at what stage of your study were you taught?

R: when I was in school of nursing, that will be maybe the second or third year, I may not remember precisely now. But it’s part of our course of study then

I: can you remember what it was called?

R: that should be which posting… it should be the gynae posting

I: okay ma. What do you know about human papilloma virus?

R: Human papilloma virus is the- actually the virus that causes the cancer and I think they are in different types. ((Phone rings))

I: what of HPV vaccine? What do you know about that?

R: the HPV vaccine to my understanding is the vaccine that we give to women or girls between- about 25 years to protect them against cervical cancer

I; are there different types of this vaccine?

R: I understand there are different types, there is a particular type that is common in this area which is the ceverix that we give to women. I understand that there is another type that they give to men and I think research is saying that a particular one we use for women can even be given to men as well.

I: what of the schedule? Is there a particular schedule for taking this vaccine?

R: yes and- the HPV vaccine normally should be given to girls from the age of 9years, below 9years they cannot have it. so the schedule for ages between 9 and 14years is different from schedule for ages above 14 to 50years plus. So for ages between 9 and 14 the schedule is they will take the first dose 0 and 6months. that is how we refer to it, that is they will take the start dose, then take the second dose 6months after. For ages over 14years they will take 3doses which 0, 1, 6. That is the first contact, a month after, then 6months after the first dose

I: now you mentioned that the vaccine is for prevention, are there other advantages of the vaccine?

R: well I think since the main- what we expect the vaccine to do is to protect them from having the virus so the work we want it to do is what it will do. The only thing- … can you ask the question again?

I: the importance of the HPV vaccine

R: the importance is just that it helps to prevent cervical cancer

I: what will be your recommendation for the vaccine in Nigeria?

R: I will recommend that it be given to every lady, girl and women that are still within this age range of having it. another recommendation is that if the government can make it available to adolescents- girls from 9 to- well within the school, from the age of 9 to adolescence, they should make it part of the immunization schedule, they should make it available for everybody

I: you just mentioned the immunization schedule in Nigeria and that is the next I will go into. If we say we should introduce the HPV vaccine into the immunization schedule in Nigeria, do you think there will be some benefits attached to that? And what will be the benefits?

R: yes there will be some benefits attached to it, since prevention is better than cure and if we prevent the disease it is much cheaper than to treat. So I think economic wise it is cheaper for the government, the money they will use to treat will be far more than the money to prevent it and that way it will improve the health status of the nation. If the women are healthy, the nation will be healthy. And at least there will be hope for the future that at least these young ones are already protected so they will not be coming down with the infection.

I: so what then can you say will be the disadvantages of introducing the vaccine into the routine immunization schedule?

R: disadvantage? Well for the people taking it, I don’t think there is any major disadvantage but on the part of the government, they may think it is costing them a lot to do it. so for economic reasons the government may be spending more but if they think of it at the end of the day they are only saving. So I don’t think there is any appreciable disadvantage.

I: thank you for that. Ma are there challenges you envisage may come up by introducing this vaccine into the routine immunization schedule? Maybe you have some concerns or challenges that you think may come up as a result of introducing it

R: if they really plan well and from onset they know what it is going to cost them and they are ready to take it up, I don’t think there should be challenges or problems

I: okay ma. Is there any reason why you will not freely recommend this vaccine for an adolescent?

R: I don’t think I will not recommend it

I: you don’t think any circumstance can come up that will make you to be skeptical about recommending it to an adolescent?

R: I will recommend it, I don’t think there is any reason why I wont

I: have you ever had to recommend it for an adolescent?

R: yes I have had to recommend it on many occasions

I; can you give me some of these instances that you had to recommend it?

R: well I am a public health nurse, I give health talk to mothers most of the time and each time I have opportunity I make them to be aware of it and I do encourage them to take it. to let their daughters take it and also for themselves.

I: what has the response been like?

R: there has been a lot of positive response; they have been coming to have it. some- most of them are happy to have the information and some of them that are not opportune to take it have the mind to take it as soon as they can afford it. Actually that is a bit of a challenge because so far the cost of taking it is high, to take the three doses or the two doses is something some people cannot afford. That is a form of a challenge, if they can find a way beyond this challenge they do find a way to come for it.

I: on a final note, what will be your recommendation to ensure adolescents get this vaccine? What do you think can be done?

R: it will still go back to the government to try and make it free if they can and even if they cannot make it free, they should find a way they can subsidize it for the public so they can have it because considering the economic situation of the country now, it is not everybody that can afford it and it is something that should benefit everybody. So my recommendation is that government should come in and see what they can do to it, so far they have been trying with the other immunization plan but they can try more by making it free or at a subsidized rate.

I: thank you so much ma, we really appreciate you.
