# Supplementary material for: Facilitators and barriers of healthcare workers’ recommendation of HPV vaccine for adolescents in Nigeria: views through the lens of theoretical domains framework
Source: BMC Health Serv Res. 2022 Jun 25;22:824. doi: 10.1186/s12913-022-08224-7 (PMC9233785; doi:10.1186/s12913-022-08224-7)
Supplement: Supplementary file 8 — Additional file 8. [file 12913_2022_8224_MOESM8_ESM.docx]

Topic: Knowledge of Cervical cancer, Human Papilloma virus and HPV vaccine

Interviewer: A

Note-taker: AA

Identifier code:

Language: English

Number or participants: 1

Date of Interview: 1/02/2017

Time of activity: 00:19:31 (hours: minutes: seconds)

Date transcription completed: 06/03/2017

Transcription completed by: A

I: good afternoon ma [good afternoon]. My name is AAAAA and I will be asking you some questions to explore your understanding of cervical cancer, HPV and HPV vaccine. I assure you that whatever you tell us will be kept confidential and will only be used for this research. Do you grant me permission to go ahead with the questions? [yes] and you agree to this recording? [no problem]. Okay ma, can you introduce yourself?

R: I am the assistant chief nursing officer, I work in a primary healthcare centre- Kola daisy foundation, it is an affiliation of University College Hospital. And I have been working in UCH- this is my tenth year. But I have had 14years of experience.

I: How old are you ma?

R: I am 39years

I: your qualification?

R: I am a registered nurse

M: okay thank you ma, can you tell me what you know about cervical cancer?

R: cervical cancer is one of the leading cancers in women, I think it is rated second after the cancer of the breast and the cause is unknown but human papilloma virus has been implicated as one of the causes. Then it also affects women who have had- who have been exposed to sexual intercourse and especially women who have had more than one sexual partner. And it has also been said that one of the factors- I mean one of the-how will I say it now, you know I said the cause is actually unknown but one of the risk factors, it is common in multiparous women. And actually it is one of the cancers that can be prevented, and if detected on time can be cured

I: you mentioned prevention, but before we go into that is there a particular age that is more at risk of cervical cancer?

R: women that are sexually active and that are also of reproductive age

I: you said something about it been preventable [yes], what are the ways of preventing it?

R: the recent lectures I have attended, they have mentioned that there is a vaccine that can be given- you know just like I said that HPV has been one of- the major virus that has been implicated in cervical cancer and because it has been found out that it is one of the causes. If women especially women of reproductive age are given this virus, I mean this vaccine it can prevent cervical cancer in the future.

I: so asides taking the vaccine, are there other measures of prevention?

R: yes, decreasing….when you don’t have multiple sexual partners, it can also be one of the ways of prevention. Then the- you know I said multiparity is also- multiparous women are also prone to cervical cancer, so reduction in- low parity can also be one of the preventions

I: thank you ma. Is there a name that people call it in this community, you know we call it cervical cancer, is there a common name that people use in this community

R: yes. You know just like the Yoruba call it ‘jejere ile omo’ that is the common Yoruba name for it

I: all these things you talked about, where did you learn it from?

R: from the training and as we come in come in contact with some of these cases, we read up on them and when we go for seminars and updates. Also during service training. These are some of the areas we learn such.

I: during your period of training as a nurse or is it as a midwife [yes] during your midwifery course, did you at any time have any course where you were taught cervical cancer?

R: yes

I: at what level was that?

R: that was my 200level in school of nursing [can you give me-] during- when we were having maternal and child health care

I: can you give me the details of the course, what did they teach you then?

R: it is just the causes, the signs and symptoms and the options of management that are available, then for those are already in terminal- then the stages of the cervical cancer, you know it comes in stages. Just as I said when we started the discussion that if it is detected early and treatment is initiated, it is curable. So we were taught the stages, I have mentioned the management then for those at the terminal stage, the palliative care that you can give to them and prevention. Then there was no vaccine so it was more of- then what we were taught more of prevention like public awareness, early detection and management. Then there were no vaccines.

I: thank you ma, you already mentioned HPV as the virus that causes cervical cancer, can you tell me what you know about HPV vaccine, the types, and schedule?

R: I don’t really know much about the types and the schedule, what I just know- I don’t know but I think they said three doses, I don’t know the actual schedule but I know that they can have it as early as maybe 19years or 13years. I think 19years, from teenage 19years and 35years. I am not too sure now, I don’t know the schedule

I: so you don’t give the vaccine here?

R: no

I: what is the importance of the vaccine? You said it is-

R: it is for prevention

I: is it given to those that are already having sex or-

R: I think it can be given to those that are not sexually active before the person becomes sexually active and she can have it.

I: what will be your recommendation as regards administering the HPV vaccine in Nigeria? Do you have recommendations on how it should be done, this is how it should be done, this is how it should not be done?

R: well for now, because my knowledge of this vaccine is not full, I don’t have a full knowledge of it so I cannot really say that it should be instituted as part of the normal schedule immunization that we have because personally I don’t- you know vaccines, they are supposed to be researched over years, the outcome and whatever side effects. And because of our environment, before you can institute a vaccine like that you must create awareness which I don’t think has been done, then you should also give room for educating- I think health workers should be well educated about the vaccine.

I: thank you ma, you already mentioned the routine schedule; do you think that it should be introduced into the routine schedule?

R: maybe but I don’t think it should actually be- I don’t think it’s really really necessary to make it as part of immunization schedule

I: why do you think it is not necessary?

R: because the knowledge about it now, just as I said that the knowledge about it now- for me I don’t even know the schedule, I think I just heard that it is 3doses and it is health practitioners that will educate the public. So when you don’t have the knowledge of something, you don’t know the side effects, you don’t know maybe the vaccine is given to someone that is not sexually active, if it can- if it may have side effects later in the future. It is very difficult now for me to say that it should be in the routine schedule for now but if we are well educated, we have the full knowledge of what the vaccine is actually- what the schedule is, the side effects, the complications that may arise, maybe if the knowledge is full and adequate, maybe I will recommend it.

I: maybe I should push that further. If we- let us say we now have the vaccine as a routine schedule, do you think there are some benefits- what do you think would be the benefits of introducing it?

R: it will be beneficial but at the same time we have to look at the financial implications. I said it will be beneficial because it will reduce the rate- the mortality rate and the morbidity rate of cancer. Then it will also make the community to be aware of what the vaccine is about.

I: thank you ma. What will now be the disadvantages? Do you foresee any disadvantage coming up from introducing it?

R: just like I said that the knowledge is not adequate enough so now the complications, side effects, everything- have not really been explained in details so [you see that as a possible disadvantage?] it is possible, there is nothing without side effects, though it is going to be beneficial but I think if we are able to work on it over a period of time, we should know what and what will come out of it.

I: generally are there some challenges that you foresee, you know we have discussed the benefits and the disadvantages and you have even mentioned some of these things, are there some other challenges that you foresee from introducing the vaccine into the routine schedule, are there challenges that could arise maybe on the part of the community, the government or the health workers?

R: I know it is not part of the vaccines that are given free of charge and you know once you introduce money it may be somehow difficult so I think one of the problems is the financial issue. Then because of our environment, ignorance…are you getting me now, when people are not educated on this thing, they will be saying what kind of vaccine is this one, who is praying for cancer. You know from our traditional belief, people believe that cancer is a punishment from God as a result of something evil that someone has done or as a result of spiritual attack, things like that. So if you say you want to start giving vaccine, people will say that this cancer is not ordinary, it is a spiritual problem so vaccine can never be a solution to it. so because of ignorance and financial implications, these are possible challenges that may come up from introducing it

I: will you freely recommend this vaccine to an adolescent?

R: ((sighs))

I:if you will, tell me why and if not, why not?

R: well for now, I may not want to recommend it for now because the knowledge- just like I told you, personally I have not had adequate knowledge about this vaccine. My knowledge about it is very shallow so I may not be able to say emphatically that I will recommend it but if I have full knowledge and I am well educated about the vaccine I will recommend it later

I: okay ma. Has there been any instance where you recommended the vaccine to someone or you have never recommended it?

R: actually I have not recommended it but I have talked about it to people not that I recommended that go and take the vaccine. You know at times people will just call you that maybe this health talk and things like that, I have given health talks about it that okay there is a vaccine that can be used to prevent it but most times I do tell them to go to our central immunization clinic in uch

I: thank you ma. Are there other recommendations that you could give if this vaccine is to be introduced and we want our adolescents to take it? Are there some recommendations that you will say this is how you should do it, this is how you should go about it to ensure that adolescents get the vaccine?

R: I think the most important thing we need to do first is to make sure that the healthcare providers or when you say medical practitioners, everybody should have adequate knowledge first about this vaccine, especially people at the grassroots. There should be enough health education for them and then you know from that area we can gradually move into the public. I think the most important thing is health education, let people know the reasons why this vaccine is necessary. And then the financial aspect, if it can be subsidized. I know it may not really be free but if it can be subsidized, then we should try and go to schools, even the teachers. Are you getting it? [okay] teachers in schools can be educated; once they are educated they can pass the information to the students. When the students get home, they can tell their parents. So apart from the health sector, they can move to schools then to the community. Gradually like that they will get a larger percentage of the public. So public awareness is very important.

I: thank you ma. That will be all my questions. Finally do you have any word on cervical cancer, HPV or HPV vaccine that you feel will be useful for this research?

R: ((sighs)) I don’t really have anything, I think that is all. We need to educate people more, that is the most important thing.

I: thank you so much for your time.
