# Supplementary material for: Facilitators and barriers of healthcare workers’ recommendation of HPV vaccine for adolescents in Nigeria: views through the lens of theoretical domains framework
Source: BMC Health Serv Res. 2022 Jun 25;22:824. doi: 10.1186/s12913-022-08224-7 (PMC9233785; doi:10.1186/s12913-022-08224-7)
Supplement: Supplementary file 9 — Additional file 9. [file 12913_2022_8224_MOESM9_ESM.docx]

**W EZ AINTERVIEW ID: NOT SPECIFIED**

**TYPE OF INTERVIEW: IDI**

**PARTICIPANT: SOCIAL WORKER, UNIVERSITY COLLEGE HOSPITAL, IBADAN**

**NUMBER OF PARTICIPANT: 1**

**INTERVIEWER: B**

**TIME OF INTERVIEW: 17:41**

**LANGUAGE OF INTERVIEW: ENGLISH**

**VENUE OF INTERVIEW: UCH, IBADAN**

**AGE OF PARTICPANT: UNKNOWN**

**GENDER: FEMALE**

**DATE OF INTERVIEW: 01-02-2017**

I: my name is AAAAAA, I am a Phd student, I am here to find out your views about HPV vaccine to adolescents in our environment, you are a medical social worker, so nice to meet you ma

R: you are welcome, My name is xxxxxx, I am a social worker, a medical social worker, I will be glad to tell you what I know, I am to learn more from you,

I: I will appreciate it, thank you very much for the opportunity ma, please ma, can you tell me what you know about cervical cancer,

R: I know that it is a kind of cancer that is second commonest in women apart from breast cancer and it is I think more in Africa than other parts of the world and I also know from my little knowledge that there are some predisposing factors, although nobody really knows the cause of cancers, that part of the reasons that could be predisposing is frequent ,I mean too, too early exposure to sex , through many sexual partners and in some women , it happens when they have had children too many times, that’s the little I know about it, and I know a little bit about the symptoms, makes a woman to bleed excessively from time to time’, because of the nature of the cancer

I: by bleeding what do you mean

R: if a woman is not having the regular menstrual period, there is really no reason why a woman should bleed from the vagina, but when this begins to happen, if there is frequency of bleeding or even any abnormal bleeding at all, it calls for attention, and also from my understanding , if the woman is bleeding during or after sexual relationship ,it can also be a warning sign and then such a woman has to do something about it quickly

I: thank you ma, please ma, can you explain what you know about the prevention of this cervical cancer

R: I know there is a vaccine, I just got to know about 2 years ago that there is a vaccine, I don’t know if it is still in existence, but I know that in Canada and in the US, there has been this vaccine as far back as 2006, this is a vaccine that should be given to young ladies, there are 2 types of it, the first one is given to a lady who has not been exposed to sex at all , who should be a virgin and she has three courses of this vaccine and that takes her for the rest of her life and can prevent her from having cancer of the cervix, the second one is for women who have been involved in sex actively, but they have to go through a kind of test and they have to be certified okay before they could proceed to having the vaccine, that much I know

I: thank you ma, have you ever heard of pap smear

R: yeah, I do

I: please ma, can you tell us, what role that can play in cervical cancer prevention?

R: well ,it is a form of prevention for women to do pap smear , I think once every two years, just to let them know their status, you know there are somethings in the body that you may not know about until they just begin to manifest in different ways, but with pap smear, a woman can screen and see the status of her womb or the neck of the womb

I: during your training, did you have any course that you went through may be on vaccine or in the process of, did they tell you about cervical cancer prevention

R: sure, we have had seminars, we have media education about how to prevent cervical cancer and this is by the pap smear screening and then just being, being totally and generally careful, because most cancers apart from cervical cancer are caused by lifestyle, lifestyle contributes a lot to predisposition to cancer

I: the seminars you were talking about, is it seminar when you were training in school

R: some while I was training in school and I have to do a pap smear by myself while still studying as part of the education I have and also

I: you mean you performed the pap smear on somebody

R: no , no , no, I had it

I: okay you had it

R: still on the job, from time to time we have reasons for education here and there, seminars, training on what to do, in order to prevent some kind of general sickness,

I: at what stage of your training were you exposed to these things you mentioned, can you remember

R: yes, I can remember, my second year for my post graduate, that was when we heard about it, most often some things are there, and you don’t know they are there because you are not a victim of this things, you are not being directly affected, you may not know about it, I did not know about, I have never heard of cancer until I was studying and then that was around 2006, I was over 40 at that time, so when I got to know about it, I was reading and any other thing that someone has to be careful about it

{phone rings}

I: thank you ma, you have told me about HPV vaccine, the types that you know, do you know the names of any one, those two vaccines that you spoke about earlier

R: I just know there is one for young ladies who are not exposed to sex at all and I know there is another one for women who have been exposed to sex{ phone keeps ringing} actively involved in sex, I may not know the medical name directly

I: are you aware of any recommendation for this vaccine in Nigeria

R: how do you mean

I: like is there a policy in place or government legislation about these vaccines

R: no, like any other thing in Nigeria, the government is careless about our health unless you are a stake holder, in your own affair, apart from, by the grace of God, I work in a hospital, I want to know about my health and I want to learn more, it was in the course of that that I went through the training and the things that we have heard, there has not been anything about the training from the federal government, you just take your own life and know what you want to do about it

I: so if the government decides to wake up to their responsibility and decides to introduce this vaccine to the list of routine vaccines that we have, will there be any advantage

R: sure, very much so, there will be so much advantage because it will help, especially if it is free, a lot of this things people know they are there a lot of people know, they can’t even afford the cost, there should be access to health care, that is what I know, because you can’t be any sane unless you are well, wellness is not just about physical appearance, it is also about the prevention , the vaccine that I know right now will cost 21000 naira for a young lady , I don’t know what it will cost for women and 21000 naira is a fortune, you know, so we look up to federal government for a lot of this things that we need, so that things can be easier and we want the federal government to make this things affordable, if it is not totally free, let them be affordable

I: subsidized

R: subsidized

I: so if the government decides to make the vaccine routine and make it free, what do you think are the advantages

R: well, one, the government will have performed her role, her duty , her responsibility like other western countries, I know health care could be costly but everywhere in the western world, health care is subsidized, number 2, it will help a lot of women, because this virus is carried by a man who comes home to sleep with his wife, neither of them has the idea that the man carries this, even the man does not know that he carries it, it is so unfortunate the way it happens and when it has happened, what do we do, if the woman has access to health care probably if she has a vaccine, she will have some peace of mind about it, then you know our culture, everything boils down to the woman, even if she knows that her husband has medical issues, she cannot every time say no to sex even if she does not like it, so if she has to be there and be well for the children, she has to be protected and this is a way of protecting our women, then number 3, it will be affordable, even if it is 10000, and she has to pay 10000 three times and she knows the value of this vaccine and she knows that it will make her healthy, she will save 10000, over this period of this three courses but 21000, will make her feel because of other things she needs to spend money on, then I, so I think we have a lot of, and when women are fine the whole nation is fine, women here, women, there, women at work , women at church will play a lot of roles, once we are healthy ,once we are fine then we are empowered, and we will be able to attend to other things and we will be able to contribute our own to the development of the nation

I: thank you, apart from these advantages, do you think there will be any disadvantage if this vaccine is introduced

R: [phone rings} I don’t think so, unless there is a side effect, if there is no side effect, I don’t think there will be any disadvantage, unless it will cost the federal government some money, which we all know

I: there are some people who have been concerned that this vaccine specifically for younger children before they ever start sexual activities, WHO recommends, 10 to 12 years, some have expressed concern that this children after giving them may become promiscuous , do you have any concern, do you have any reservation about it

R: I don’t think so because it is not a family planning vaccine , it is not a family planning stuff and also everything depends on education, education empowers, you know if you tell a child, this thing you are doing is for so, so, so purpose, she will know and she will have it in her head, health education is very, very important, if they make it happen, it does not stop anyone who wants to be promiscuous, if they make it happen, there is more advantage in that they will know that they are protected but they don’t have to be exposed too early because you still keep guiding them, you still tell them about our culture, you still teach them values, the society values, I don’t think, I don’t see the correlation, it is not a family planning method

I: should the government decide to introduce this vaccine, do you envisage any problem or challenges in the process of introducing it in this country

R: I don’t think so because it will only cost some money, it will make the federal government to cough out some money, which we have , which we can afford especially, if it has to do with medical care, I don’t see any reason why we should not be able to afford it,

I: so if the vaccine is introduced into the routine schedule, will you feel free to recommend it for people who it is meant for

R: sure, I have heard about it, again and again and so far, I have not heard anything about the side effects, unless you know of any, I will recommend it gladly because it will prevent a lot of havoc in the future

I: then have you ever recommended it for anybody may be a friend

R: yes, yes, I have recommended, I have talked to several people about it, and I still keep talking about it

I: which kind of people,

R: young ladies, especially, I have not done it myself, but I have introduced it to some people, I have talked to people about t

I: have you ever had any cause to talk to , may be parents about it, for their children

R: because I have a youth program from time to time, I have only spoken to youths about it, I have not really talked to parents about it

I: youths like what age range,

R: Like teenagers, let’s say teenagers, like my team players, we have a lot of things to do together,

I: like how many tim]es have you had to do this together

R: well I won’t , I may not be able count the times, but as many times as I have to do things with them, especially when it is about medical, I tell them they need to do the test, I tell them all they need to know about it I encourage them, like I said it is expensive,

I: what of in hospital setting, have you had any cause to talk to adolescents about this

R: adolescents, yes, but not parents

I: not parents, thank you so much for your time, I really appreciate it,

R; thank you Dr Balogun
