# Supplementary material for: Facilitators and barriers of healthcare workers’ recommendation of HPV vaccine for adolescents in Nigeria: views through the lens of theoretical domains framework
Source: BMC Health Serv Res. 2022 Jun 25;22:824. doi: 10.1186/s12913-022-08224-7 (PMC9233785; doi:10.1186/s12913-022-08224-7)
Supplement: Supplementary file 10 — Additional file 10. [file 12913_2022_8224_MOESM10_ESM.docx]

**INTERVIEW ID: 170222_002**

**TYPE OF INTERVIEW: IDI**

**PARTICIPANT: HOME VISITOR, UNIVERSITY COLLEGE HOSPITAL, IBADAN**

**NUMBER OF PARTICIPANT: 1**

**INTERVIEWER: T**

**TIME OF INTERVIEW: 16:01**

**LANGUAGE OF INTERVIEW: ENGLISH**

**VENUE OF INTERVIEW: UCH IBADAN**

**AGE OF PARTICPANT: 56**

**GENDER: FEMALE**

**DATE OF INTERVIEW: 22-02-2017**

I: good morning ma, my name is XXXXXXXXX, I am here to find out your views about cervical cancer, human papilloma virus, and human papilloma virus vaccine, before we continue, I will like to seek your consent to go on with this interview,

HVISITOR: okay, approved

I: thank you ma, and your permission to record this conversation

HVISITOR: please do

I: thank you ma, before we continue, I will like to know a bit about you, what your work involves, how old you are and some other information

HVISITOR: I am a health visitor, and health visitor, means I go on home visits, and I disseminate information especially in paediatrics unit to mothers, about their children and adolescents, about their health , the health way to bring up children , so that they won’t have infection in the nearest future, they will be infection free

I: how long have you been working ma

HVISITOR: I have been on it for past eh, can I say 20 years now,

I: that’s a long time, how old are you now

HVISITOR: hmm, I am 56 now

# Q.1. Can you tell me what you know about cervical cancer?

I: thank you very much for the information ma, please ma, can you tell me, what do you know about cervical cancer,

HVISITOR: cervical cancer, as the name implies is cancer of the cervix, it has its own signs , its own symptoms, if it is not treated, if there is any coitus, during coitus, there could be bleeding, there could be erosion of , you know erosion of the cervix, yeah , bleeding is the major issues, that’s why some people don’t detect it early because the bleeding might come much later, it is later, you know, that’s what I know about cervical cancer

# Q.2. Please explain what you know about cervical cancer prevention.

I : do you know how it can be prevented?

HVISITOR: I know through vaccine, immunization, you know, Cerverix, If I pronounce it well, vaccine can be given , I think from 9 years of age adolescents through, after 40, you can still have it in divided doses, you know, the ideal time , I think its about 9 years of age,

# Q.3. Describe what you were taught about cervical cancer prevention during your training

I: thank you, have you heard any training related to cervical cancer at all

HVISITOR: no, I have not had any training at all

# Q.5. What do you know about HPV vaccine?

I : ma, the vaccine, Cerverix that you said it comes in doses, do you know the schedule of the vaccine,

HVISITOR: no, no ,no, I am not conversant with the types, you know, I don’t know, for that kind of vaccine, for an ordinary new person at home, I won’t be telling the person that we have type, this is the name, all I will say is that we have immunization, preventable for your adolescent girl child or for yourself as a mother to prevent cancer, and cancer as the name implies could be very, very dangerous, that’s what I will tell the mother to just prevent it but I don’t know other names because I am not in the pharmaceutical

# Q.6. What would be the benefits of introducing this vaccine into the routine vaccine schedule in Nigeria?

I: ma, can you tell us the importance of the vaccine, what do you think is the importance of the human papilloma virus vaccine?

HVISITOR: well the vaccine is, you catch it early and it prevents and even when its ongoing, erosion or erodes of the cervix or there is an infection, you can still take it and it will still you know, help, help in curtailing the spread of the cancer at that time,

I:ma, the vaccine, if its introduced into the national schedule of immunization that runs presently, what do you think will be the benefit

HVISITOR: the girl child will be cancer free, let me use that word, cervical cancer free, even at school , you might tell them you still have this immunization, it does not have to end at nine months or whatever, it can be ongoing nine years may be some booster doses at intervals, so, to prevent and reduce the influx of cervical cervix, and that’s the preventive aspect, you know, health information will be in, promotion health promotion , you know, prevention is better than cure

# Q.7. What would be the disadvantages of introducing this vaccine into the routine schedule in Nigeria?

I: ma, do you think there may be any disadvantages if the vaccine is introduced

HVISITOR: well, for many other drugs, I do know there are disadvantages, I don’t know the side effects, for some vaccines when you give it, they will tell the children , I mean the mothers to give some paracetamol, there may be reaction, some are live, some are attenuated vaccines, so I don’t know what the preparation is, some may be, I don’t know if there are some who may still react to the vaccine, or not react to it, it depends on the property of the vaccine, but I know that there must be side effects at least

I: so apart from the side effects, you don’t see any disadvantages of the vaccine

HVISITOR: I don’t, If it is, I think its preventive, its prevention, there shouldn’t be any disadvantages, advantages should be more, at least, if not eradication of eh cervical cancer, catch it young, from nine years and above, and they are aware that they have to take the doses to certain age, I think, even the elderly , not even the adolescents alone, people that are above the age of adolescence they can still take it, so that if they see any sign of bleeding during coitus, they should just report to the hospital, that’s all I know about the vaccine,

# Q.8. Are there any challenges that you envisage may arise should the vaccine be introduced to the routine immunization schedule in Nigeria?

I: ma, what do you think will be the challenges if this vaccine is introduced, challenges, when I say challenges, I mean , human resources, finance, all those things,

HVISITOR: right away, availability of even the normal vaccine, the normal BCG, at times the mother will tell you, it is not in their health centre, go to odogbo, availability , is it affordable, is it accessible, is it something you can just go to health centre, or vaccine centre and take, or do you want to take to the school children, can you carry it to the schools and inform the principal that today is a vaccination day for cervical cancer, age so so so, even in primary school, some of the are still nine years, so affordable, is it, I don’t know the price, with this economic recession, is it available, then the cold chain, I don’t know whether it uses cold chain like any other vaccine, I don’t know the property , the cold chain, will It be effective after taking it, will somebody still come down with cervical cancer, so those are the challenges, then the man power, how many people will be going to, because I believe they should be going home to give the vaccine, after jingles, is not BCG alone, because this vaccine is pretty new, not many people, let me ask you , do they take vaccine for cervical cancer, is there any thing called cervical cancer, some will tell you, traditionally, they won’t even believe there is cervical cancer, they will be like, is there anything like that, so they have to go to schools, to mosques, to churches, to everywhere, we don’t even have the manpower, federal government , I am sure will not be able to afford it, paying any money and ordinarily, people on the wards will not be going out too, to go and do jingles on it, I mean passing information, salary payment , all those things, manpower, then you talk of money, you know, where is the fund, is it from UNICEF, is it from united nations population fund, who is funding it, that’s the main question, who is in charge , will they divert the fund like any other one in Nigeria , that they used to do, and then when you talk of the materials, the syringes, I am sure they will use syringes , needles, cold chain, fridges, reservoir, I don’t know, so where are they going to get, all these, you know, 3Ms, money , material and man power, I hope its not going to be an obstacle, and then ,you need to know, that people will not divert money, people will not be taking it to their clinics, instead of giving the populace, so those are the things you should consider, these are the challenges we have in Nigeria, with ordinary vaccine, with ordinary BCG that everybody knows about, so I don’t know how they are going to challenge it

I: ma , what do you think we can do to, all these challenges you have mentioned, how can we surmount them

HVISITOR: I don’t know whether ,our primary health care are still functioning, because the health minister mentioned that the primary health centres are going to be functioning, if its functioning as he said, I am sure, we can work into primary health care centres, nearest to your house , availability, or I don’t know if they can even bring it to tertiary hospitals, like UCH, state hospitals ,that is secondary , hospitals how, they could access it, and they should give it to people that are well trained , not just anybody, like the institute of child health, and trust, put the trust on them, that they will carry out , otherwise, it will go down the drain like any other vaccine

I: do you tell them about the cost

HVISITOR: I don’t know the cost, because I don’t deal with immunization at all, its just to tell them about immunization, I just tell them to take the vaccine, I will tell the parents, that there are so many immunization they are supposed to take, for BCG , I don’t give immunization, but their awareness, my priority will be exclusive breastfeeding for the next six months, if I have a baby , this is what they are going to do, but afterwards, I will tell them about immunization, prevention is better than cure, I don’t handle immunization , so I don’t know,

I: ma, considering your position as a community person, the vaccine is 8000, do you think that if you take the vaccine, and you tell them that for each dose , it is 8000, and they have to take two or three doses, what do you think will be their reaction,

HVISITOR: that’s, with this economic recession, I think it’s pretty high, honestly, but then , the cost effectiveness of vaccine , not that I am going to say it’s too much or not, we have to weigh it together, you will let them appreciate that , what you are going to spend on treatment if the girl is down with ca cervix, what you are going to spend is even more than 10 times over, and at the end of the day, there may be spreading, metastasis, so it’s even better, to have this vaccine at all cost, but they will resist it at first, I don’t know what the government can do for them ,so that they can now get it at a lower price, but 8000, I don’t know, it’s like it’s for elites, people that have money alone, because a market, if you go to market now, that there is one vaccine ,that is preventing cancer, everybody will be happy, to receive it, but they will ask you, how much, 8000, ah, the price is high but then the gain is much, so we should work harder and let them know and then the government can even come in and subsidise the rate of it as well

# Q.9. Is there any reason why you will not freely recommend the vaccine for adolescents?

I: thank you ma, have you ever had to, is there any reason why you will not want to recommend this vaccine to an adolescent,

HVISITOR: I will recommend it, I will let them know, when we go on home visits, we usually tell them, we tell them there is a new, I will use new, it’s not new per say in other developed countries, I will tell them, people have not been taking it per say, that this vaccine, is reliable, its good, let me use that word in quote that they should take it, to prevent

I: thank you very much ma, I don’t know if you have any other, in addition to everything we have discussed

HVISITOR: nothing, except that there isn’t much information about, it’s not yet, not many people are aware about, government and even the people,, let them know from schools, we call it school health services, and let them know, it’s not as if they want to give them when they get to school, but awareness, you are in school, you will need it , so tell your parents, market place, there should be jingles, radio , television, and create an awareness for everybody, if they are really aware, they will be willing to take it no matter the price, because the price is nothing compared with the illness, and the amount of money they will spend you know terminally , for such an infection ,that’s all I have

I: thank you very much for your time ma, that will be all
