# Supplementary material for: Facilitators and barriers of healthcare workers’ recommendation of HPV vaccine for adolescents in Nigeria: views through the lens of theoretical domains framework
Source: BMC Health Serv Res. 2022 Jun 25;22:824. doi: 10.1186/s12913-022-08224-7 (PMC9233785; doi:10.1186/s12913-022-08224-7)
Supplement: Supplementary file 11 — Additional file 11. [file 12913_2022_8224_MOESM11_ESM.docx]

**INTERVIEW ID: 170217_001**

**TYPE OF INTERVIEW: IDI**

**PARTICIPANT:FAMILY PHYSICIAN , UNIVERSITY COLLEGE HOSPITAL, IBADAN**

**NUMBER OF PARTICIPANT: 1**

**INTERVIEWER: T**

**TIME OF INTERVIEW: 32: 37**

**LANGUAGE OF INTERVIEW: ENGLISH**

**VENUE OF INTERVIEW: UCH, IBADAN**

**AGE OF PARTICPANT: 42**

**GENDER: FEMALE**

**DATE OF INTERVIEW: 17-02-2017**

I: good morning ma, as I said earlier, my name is XXXXXX and I have your permission to go on with this interview and record this conversation, but before we continue with this interview, I will like to know a bit about you, like how long you have been working as a family physician

R: I have been working as a family physician since 2009

I: okay

R: that makes 8 years

I: how old are you ma,

R: I am 42

I: and you have been working since 2009, thank you ma, can you tell me what you know about cervical cancer

R: it is a kind of cancer found in women and it affects mainly women in the reproductive age group and even women in advanced age depending on the onset of the disease, it is ehm, it is a cancer of the mouth of the womb, it is ehm, it is ehm, there are symptoms that can occur, it passes through some stages,pre cancerous stages, which we call the cervical inpacteria, neoplasia, that is it can be picked early , it is caused by a virus, human papilloma virus, specially strain 16 and 18, which is preventable, it is sexually transmitted, when there is inflammation in the mouth of the womb or the cervix, it now undergoes neoplasia, it is not addressed at that time, it can progress into cancer of the cervix, it takes some 10 , 15 years before it becomes frank, cancer of the cervix

I: so it takes about 10 to 15years

R: yes, from the precancerous stage to the cancerous stage

I: thank you very much ma please ma, can you explain what you know about how we can prevent this cancer, how can it be prevented

R: because cervical cancer as I said is a preventable cancer, and because the organism that causes it is a virus, that is the human papilloma virus, which is transmitted sexually, so if that chain can be broken ,by being faithful to one’s partner and use of barrier method to prevent the transmission of the infection but one major thing is also by regular screening to detect the pre-cancerous stage and its treatment so that the person does not develop the cervical cancer, and then there is also the option of the vaccination which one can take, especially among the adolescents, I mean, between the ages of 9 and 26, though studies are ongoing and it shows that after the age of 26, people develop immunogenicity, so with the vaccination of young girls and young women, the virus is prevented and the person obviously will not develop the cancer of the cervix ultimately

I: thank you very much ma, this information you just gave me, how did you come across it? how did you get it

R: I am a doctor, of course part of our training is learning about cervical cancer, the causative organism, the prevention and treatment and so on and so forth, and apart from that , I have gone for conferences where it is being discussed and I have also read some pages of books, and then gone for some seminars that held

I: okay, so apart from the regular class, you have also had to go for some conferences, [yes, yes] okay ma, thank you very much, please ma, can you explain what you know about the virus, the human papilloma virus

R: the human papilloma virus, like I said there are so many strains, but the one that causes the cervical cancer is the 16 and 18 strain, the 16 and the 18 , they are the ones that are commoner, it can be carried by both the man and the woman and if the woman , I can’t remember may be its an RNA or a DNA virus again, but if a woman has it, and has sexual, unprotected sexual intercourse with a man, the man carries the virus, and then the man can have an unprotected sexual intercourse with another woman who does not have the virus, and then the woman can also catch it, that’s why when there is a sexual network, it can cause the spread of the virus,

I: thank you ma, we know there is a vaccine, ma please can you shed more light on the vaccine

R: the human papilloma virus, the type that I know about, there is the Gardacil, which covers more strains of the virus and it can even be used in males, in young boys, so that they don’t carry the virus and when they don’t carry the virus, they can’t transmit it to another, to a woman and all but when there, the one that is available in Nigeria is the Cerverix which is against the HPV, that one is against the 16 and the 18 strains

I: the cerverix

R: so it is not used in men but it is used in women, because women manifest it more

I: do you know the schedule of the vaccine

R: before a young girl clocks the age of 15, they are usually given just two doses, the first dose, start up dose, and then a month later, you get the second dose, after the age of 15, the woman takes 3 doses, the first dose, then a month later takes another dose, and then 6 months later, 5 months later takes the last dose, so it covers the span of 6 months,

I: and the immunity is for life

R: for now, yes, though studies are ongoing but that is what we have for now,

I: ma, please can you tell us, what is the importance of this vaccine, how important is it

R: well, the vaccine, like I said, cervical cancer is the commonest cause of, well, it is only in breast cancer, they are the commonest causes of cancers in women but there is one that is preventable, like cervical cancer is preventable, breast cancer is not preventable, per say like that, it can only be detected early , this will prevent it from later occurring, so the vaccine, I think the question is the importance of the vaccine, the importance of the vaccine is to prevent a woman even from having that virus, that can now predispose, that is why I said it is preventable, if you prevent a woman from having that virus, then the woman does not have the opportunity of going through the precancerous stage , talkless of going into cancer of the cervix, so why not, treat our women early, women go through a lot of stress, they have children, they have to take care of them, most women are career women now, and then at the prime time of their age, they are forty , fifty, they suddenly discover they have ca cervix, it is not the best, so why not prevent it by giving the vaccine among the young girls now, and then nowadays, adolescents and teenagers they are quite exposed early compared to those days, there is a lot of social media, on the television and then they want to behave like the westernised world, so sometimes you see them, there is early coital, they have sex early and this is one of the things that predisposes to cancer of the cervix, they have sex earlier than those days when there is dignity and virginity was the top thing among young girls because most women wants to marry as virgin , it is not like nowadays that , you are a virgin at 19, that means you are such a … or what, it is in quote not socially acceptable among the adolescents now, which is what we are trying to advocate, prevent them, stop them from having, thinking like that, that women should still, women should still maintain abstinence till marriage, I mean, giving the vaccine, I mean , if you can’t stop them from having sex early, you can prevent a disease from occurring in their prime time, when they now become young mothers, they now become middle age, they start having cancer of the cervix, it is not the best, it is the time that, even if, it is the time that they are in the work force, you need them to improve the economy of the country, you need them to, a house, a home without a mother it’s actually, it is, you don’t have the best for the children, [hmm] even if the man decides to remarry, of course there is a step mother and the step children issue and all of that, and then they become a dysfunctional home, so why would you want to , want a woman to die early, from a preventable death , it is not worth it, if it’s something that is not preventable, it is different but something preventable, why not prevent it by giving the vaccine

I: thank you very much ma, ma, what is your recommendation for the vaccine in Nigeria

R: my recommendation, because currently now, the vaccine is quite expensive, we are talking about, the last time I heard about the vaccine ,it was 7000 naira, per dose, though the timing is staggered, one can try to meet up and make up the 7000 naira before the next dose, and so on and so forth but if it can be made free or at least, cheaper, affordable, for a regular Nigerian woman, it will be very good, Nigeria mother to give to the child and then we are talking about immunogenicity being developed even after the age of 26, so women in their thirties can also have it and then, and then if it can also be included in the NPI scheme of vaccination, that will be really good, and then, the advocacy should be on the air , television, radio, in schools, the vaccine can be taken to school like they do for polio , measles, they can have days for vaccination against HPV especially among the young girls, the older women they are already sexually exposed, there may be need to screen them though they are not the carriers and they have not developed the precancerous stage and all that , so that they can also be vaccinated,

I: I wanted to ask that, you mentioned people that are older than 26, that what if they are sexually exposed

R: if they are exposed, though it’s not readily available now, one can screen for HPV but the last I heard , there is a place , a government outfit in town that they can do the HPV screening, so they can screen and get vaccinated, though at this time now, we do pap smear for them and if the pap smear is normal, they can get vaccinated but those kind of women have to still be doing their pap smear from time to time, every 3 years if it’s normal but if it’s not normal then they do it yearly till it becomes normal and then they can progress to[ even with vaccination?} even with vaccination for those that are already sexually active, because you are not even checking the HPV first and then the immunogenicity is actually better between the age of 9 to 26, so those after the age of 26 , you still advocate that they check their pap smear, because one thing is there is a difference between vaccination and immunization, if you have been vaccinated and then they did not develop the immunogenicity to it, they will go on with this false sense of security that they are protected against HPV, they are having indiscriminate sexual exposure and all that,

I: thank you very much ma, another thing you mentioned that I will like to ask further to know what benefit it will bring , you mentioned that it should be included in NPI scheme of vaccination, what do you think will be the benefit of introducing the HPV vaccine

R: the benefit of introducing the HPV vaccine is that, it’s actually, it’s ehm, it’s one of the vaccines that is supposed to be given under NHIS, though may be because of cost, most people don’t include it, but it is in the operational guideline for NHIS, the benefit of including it in the NPI is that when mothers take a vaccine and they know that it is one of the things required, it gives a mother a sense of obligation that I need to vaccinate my child, the next appointment is when this girl is 9 years old, this girl is nine years old, that sense that this is, mothers have take NPIs as what is required, what they should do, so when you include it, it makes them know that it is not an option, they know that this is one of the things that you should do for your child, it makes them to participate to have it done , rather than, I don’t have money , you know and that’s all, when we talk about prevention, prevention of cervical cancer also involves having a regular pap smear screening, which is what is, even among health care professionals unfortunately a lot of health care professionals don’t even do their pap smear, [ that’s serious] I mean, it is a procedure that take just about 20 minutes, 10 , 20 minutes, you do the procedure and then they check it under the microscope and you get the results, results takes some 3 to 6 weeks now but it can actually be done earlier, the reason for that long time, is because of the number of slides that they need to look into, but really it’s something that can be done, as you look through the pap smear, you get the result almost immediately after

I: why do you think people are not doing the pap smear or are there other forms of screening

R: the forms of screening that are available right now, and affordable is the pap smear, but people don’t do it because, they are not knowledgeable 1, I mean for the regular person but for the health care workers , sometimes they don’t remember, so they, it takes the effort of sometimes the nurses, like in the staff clinic now, we include it in our health talk and then we have some places where we put it, have you done your pap smear in the last 3 years, if not ask your doctor, sometimes we don’t remember that the major thing,[ respondent coughs] [sorry ma][ cough continues] that sometimes some people get discouraged because of the long time it takes to get the result, but if there is a means of reminding people like you can attach it to their birthdays, do your birthday this year, and do the pap smear, then next year, okay , I did it last year, then the following year, I did it last year, it’s my birthday again, it’s been quite sometimes I did my pap smear, then they remember, a lot of times, it has to do with the among health care workers, with them remembering , among non-health care workers, you find that they don’t even know about it a lot of times, and when they know about it , they don’t know, how they can access the services, am I going to go to the doctor, and another thing is that, a lot of people patronise private hospitals, If the private hospitals, the personnel there can be trained and retrained, to take the pap smear and have a centre where there will be pathologists to view the slides, then that can also make more people to access the services,

R: ma, apart from pap smear, are there other forms of screening against cervical cancer,

I: no, it’s pap smear, except the person now develops frank cancer of the cervix , that’s when you see it and then you do, biopsy , biopsy and all that , ultrasound and so on and so forth, but HPV is also a means of screening, screening for the organism that causes it, taking the…….[in audible] against HPV , that’s the means of screening and its not readily available and where it is available , may be its not affordable for too many people,

I:ma, do you think there is any disadvantage if this vaccine is introduced into Nigeria’s scheme of immunization or are there concerns

R: the only concerns which I also thought as a mother , will it make our young girls have this sense of security that I am vaccinated against this virus so I can do anything I want and all that, that may be a major concern, and sometimes occasionally, there may be some reactions to vaccines

I: okay, like the side effect

R: like the side effect, like the surroundings that are making the vaccines, and the things that are used in making the vaccines, some people react to it

I: so what can be done, will that stop them from being vaccinated

R: it depends, if it’s just a localised thing, then it’s something that can be treated and they continue with the vaccine, but if it’s a major reaction, it might be a problem, then you tell the person to abstain, to be faithful, use of condom, not to have other partners and so on and so forth,

I: thank you very much ma, ma, is there any reason why you will not recommend this vaccine to an adolescent

R: I will definitely readily recommend it! And I do recommend it to adolescents, although the cost, though I will still recommend, it’s now dependent, recommend and encourage the, [ respondent coughs] recommend and encourage the mother to take it for their children, of course , if they don’t have the money, you can only encourage, how many people will I pay for,[ laughter]

I: so what can we do about the cost, though you mentioned that we

R: if the government can really, I heard that the, which I am also a member, may be not too active as a member, there is a medical women association of Nigeria, the Oyo state branch, they plan on going to schools to give free vaccines

I: that’s great, how will they fund it

R: through NGOs, contributions from individuals and so on and so forth, MWAN is a national body, medical women though the association in Nigeria is a national body, international

I: 14,000, that’s a lot

R: it’s a lot, I mean if they are over 15 years of age, you give 3 doses

I: that’s 21 000

R: but I believe , like the hepatitis b, the double dose vial can be given as 1000, 1200, meanwhile the single dose is given at 2700, the HPV vaccine dose available now, is given at 7000, if it’s possible to get talk to the company and get them to give multi dose vial, you can still have it at a cheaper rate than how it is now, so you know, that’s how it works, that will reduce the cost , if the company, I mean they can talk to the main pharmaceutical company to make it multi dose vial , so when they give it, they give it to 10 children at once, that reduces the cost implication

I: ma, can you give instances where you have given this vaccine to patients

R: like myself, I have taken it, I just completed my dose about a year ago

I: aha,, that’s great,

R: but I don’t have a means of checking the, I guess I should be able to check, in some special labs, I don’t know, but to check the immunogenicity whether it picked up immunogenicity to it, instances have shown that people have taken It for their young girls, that are still, that are not sexually active, those that are sexually active after the age of 18 years, they do their pap smear, after the age of 21 years, pap smear starts from the age of 21, among sexually active women, so they do their pap smear and once it, people I have recommended it to, some have been able to do it, for my girl, unfortunately, availability has been a challenge but I hope that today, I will be able to start off, though she’s been saying mummy I don’t want injection and so on and so forth, you know, when they are younger, they take the vaccine but now that they are older, [ laughter] so the injection may be one of the things that discourage teens from having the vaccine, especially the younger girls, the older girls they know the purpose of this thing but the younger girls, I don’t want injection, I don’t want injection, that kind of thing, she is almost 15, so I want to give her the two doses so that I don’t have to go through the 3 doses but most people I have recommended it to , they have taken it, some because of cost, some believe that my girl is not promiscuous now, so why would I , why would she but you never know the kind of man, she is going to fall into his hands , I think, if he is the guy that has gone from one person to the other, I have seen a doctor that developed ca cervix before and all of that , as a doctor, she does not have time for social life, has been faithful, what of the husband, you never know what , even while they are married, I mean we have situations of extramarital affairs and all that, so now people talk about HIV, people also look at it, I don’t think this girl should be infected, she looks like a good girl[ laughter] it still happens, unfortunately

I: ma what can we do, you mentioned some things, apart from the cost, like the fear of injection and all that, is there a way you think, do you have any recommendation about that

R: about the cost

I: about the injectable, about it being an injection

R: oh, am, I don’t think it can be given orally, I don’t know, I haven’t heard of, like OPV, I don’t know , I don’t know of any, of course you never know what can be developed subsequently, the main mode of administration is, injectable, so sometimes, you just encourage the young girl and all, most times, the girl just needs to be encouraged, and talked to in a nice way, so that they don’t struggle, and then using a smaller needle, although its prefilled, so it has its own needle that comes with it, just basically, the pain is not that much, and then if you tell them that you too, you have had it, then they are more likely to comply

I: thank you very much ma, do you have anything to say, in addition to everything we have discussed

R: I always tell people that prevention is better than cure, if you have managed a woman that has cervical cancer, going through some surgery, though some cases that is so advanced that they can’t do the surgery, through radiotherapy, the side effect of radio therapy, I have had a woman who had the two limbs so swollen that she could not walk with them from the side effect of radio therapy and all that and when you see women suffering for something that could be preventable, I think we really need to talk about it, to increase the awareness among women, it can even be sent as this WhatsApp messages, this group messages, a lot of people do a lot of these things, they put their banners, even banners, I mean the bill boards that are on the roads, how many times have we put something like that, these are some of the things that people see it, over and over again , hearing it on the news, may be adverts, between news, even radio adverts, it will improve the awareness, even among us as health workers, we should also be, try as much as possible to improve the awareness, amongst health workers and mainly among non health workers,

I: so if they can do all this, it will make the

R: and then, making the vaccine to be cheaper, cheaper

I: how about availability, is it readily available?

R: well, in UCH where I work, its readily available, but the cost is pushing people away

I: aside UCH, have you heard of it being available in some other places

R: hmm, it’s something, the , the, private hospitals can also, just like the way we have drugs, there are drug reps that distribute all these drugs all over, I haven’t actually asked if some places have this HPV, but I know, local government I am not too sure whether they have[ that’s the PHC] yes, because it is not included in the schedule of immunization, and then , most people that usually patronise the PHCs, they are, they are not too, most of them, I mean we have people that are in the high class, high, middle class I know, we don’t have a majority of them that may be able to afford it, so that it does not expire before they are able to give the people, but any health centre, any, women can also go to health centre, and request for it and I am sure they can get a way of getting the rep here, the drug reps to get the vaccines for them on demand in this kind of centres, private hospitals too, on demand, may be for now, because when we increase the awareness, there will be more demand, because more people will be asking for it, at the long run a private hospital owner will not be afraid of it expiring before the use of this drugs

I: Okay ma, thank you very much for everything you have said, we really appreciate your spending time with us,

R: I hope it has been helpful

I: yes, it is
