# Supplementary material for: Facilitators and barriers of healthcare workers’ recommendation of HPV vaccine for adolescents in Nigeria: views through the lens of theoretical domains framework
Source: BMC Health Serv Res. 2022 Jun 25;22:824. doi: 10.1186/s12913-022-08224-7 (PMC9233785; doi:10.1186/s12913-022-08224-7)
Supplement: Supplementary file 12 — Additional file 12. [file 12913_2022_8224_MOESM12_ESM.docx]

**INTERVIEW ID: 170217_002**

**TYPE OF INTERVIEW: IDI**

**PARTICIPANT: FAMILY PHYSICIAN MALE, UNIVERSITY COLLEGE HOSPITAL, IBADAN**

**NUMBER OF PARTICIPANT: 1**

**INTERVIEWER: T**

**TIME OF INTERVIEW: 36:30**

**LANGUAGE OF INTERVIEW: ENGLISH**

**VENUE OF INTERVIEW: YEMETU, IBADAN**

**AGE OF PARTICPANT: 50**

**GENDER: MALE**

**DATE OF INTERVIEW: 17-02-2017**

I: good afternoon sir, my name is XXXXXX, and I am here to find out your understanding, I want to know your understanding and your knowledge about cervical cancer, HPV and HPV vaccine, before I continue, I will like to ask your consent to go on with this interview

R: it’s okay

I: thank you sir, do I have your permission to record this conversation sir?

R: you can record it

I: as I said earlier, I want to know your understanding and your knowledge, sir, I will like to know a bit about you, like your working experience, length of time you have been here and what is your position here and how it deals with the target group

R:well I am[ you don’t need to mention your name sir] I have been working here as a consultant family physician since 2012, and eh, I have some contact with the target population, usually whenever they want to get immunization, we send them down to our immunization clinic down there, as a consultant, we work in direct collaboration with the health workers, the public health people that are in charge of immunization, and the immunization clinic is part and parcel of the family medicine department,

I: thank you very much sir, how old are you sir

R: I am 50 years old

I : thank you sir, I will be going straight to the questions now sir, I will like to know first, what you know about cervical cancer?

R: cervical cancer, well, cervical cancer is a type of malignancy that affects the women , especially the womb, we call it the tip of the womb, you know, it is a form of malignancy that affect, it is commoner in women of child bearing age, 20, 30 , 40 years old, that is the range that it tends to affect, and occasionally too, it could affect postmenopausal women too, you understand so, and the major contributory factor that has been implicated in all this, is the human virus, it is about, it is a very, it is on the increase and what is being advocated for them, human papilloma virus, give vaccine to help abort the development of human papilloma virus, there could be a considerable reduction to cervical cancer

I: sir, what are the predisposing factors to cervical cancer

R: that’s what I have actually told you, usually malignancy, that’s what I said, nobody actually knows the cause of any type of malignancy, but you have contributory factors, so the aetiology that has been actually advanced for that cervical cancer, is that human papilloma virus, but you have a lot of contributory factors, for instance anything that causes erosion of the cervix, cervical erosion, could lead to cervical cancer, well there is, like I mentioned the other time, post menopausal, there is a lot of imbalances, that one could predispose, also some people have advocated having multiparity and all of that, that is a cause of cervical cancer, that’s one, the major growth that that one tends to have is the fibroid, that one is commoner than cervical cancer, but what matters most is ,anything that causes irritation along the cervix, can actually cause probably a mutation of the cervix, which can now lead to cervical cancer, so those are the things I can say,

I: thank you very much sir, how can it be prevented, how can cervical cancer be prevented

R:I already told you some risk factors, avoid the risk factors, some people have said that probably some sexual behaviour, could lead to cervical cancer, how true is it, we don’t know, the truth is that there is always a when someone is involved with too many partners, you understand, so you try as much as possible to avoid the issue of having premarital sex, you try to avoid it and then you try to reduce contact, then secondly the issue of human papilloma virus, one can be vaccinated , you can have vaccination against it, then also ,routine screening when someone is actually above 30 or above , you want to do what we call pap smear, you can also use that in preventing it, the issue of vaccination ,it has been advocated that you vaccinate young girls, far far below, the age of reproductive age, so If you are looking at the ages 15 to 45, in some clime , 15 to 40, so you are targeting vaccinating the person before the age of puberty, before entering the reproductive age, so you are looking at the age of 10 to 13 to 14, adolescents, years, before 18 years of age , so you are targeting the person, so those are things that you do to prevent cervical cancer

I: thank you sir, you have given so much information, sir, the information you just gave me, how did you come across the information,

R: well I told you in my line of duty, then the little I know, I have to search for it, in medical school, they teach you a lot, but you only use probably one percent of the knowledge you have that they pump into your head in seven eight years in your actual work life, but the thing is that apart from that, as a form of routine development, you have to go to other sources, look for information, attend conferences and workshop. And then in the course of training too, you have to do some , may be some little research work, and as a family physician , you have to have a broad idea over issues

I: sir, have you had a specific training that was really targeted at cervical cancer

R: no

I: well, we used to, we have had some inhouse training, we call it continued medical education, either through the residents training that we have trained, or you have a consultant presentation, at least in our area here, routinely, we clinical presentation, one of us, one of my colleague who is a consultant family physician too, that is his area of special interest, you do pap smear, you understand, if you do pap smear , you understand, in the clinic, he use to run the clinic, for that cervical screening pap smear is like every Wednesday, so from that, you can get some of the latest information,

I: thank you very much for this information, I will like to know, I want you to shed more light on the human papilloma virus, what exactly is it, what is the virus, how did it come about and all that, I want to know more about it

R: well, human papilloma virus, I cannot say now whether it is a RNA or a DNA virus, one thing that one needs to know is that, the human papilloma virus, is in the cervical environment, being a virus, when you take it out of a living medium, they are like a crystalline particles, it will look like they are not living at all, once they enter, into a, I mean when they come in contact with any living tissue, especially tissues that are subjective to their own pathogenic ehm, ehm, ehm, content, you understand, they enter, once they enter into their nucleus, and then they integrate themselves into the nuclear particle and then duplication comes in, you understand, and usually when they do like that, they first of all replicate themselves, and once they replicate themselves, they are now produced as probably part of the secretions of the chemical component of the cell in which they are, because of the way they are, their size , their size is so tiny, they are measured in microum, or micro, you understand, 10^-6^_,_ something like that , you understand, so they are very , they are very small, compared to the human cell, so they enter, they integrate and reproduce themselves, and once that cell, I mean the dissolving, they live on the nutrient of the cell and once that cell, gets destroyed, now that virus, they are released in thousands, to affect other cells, so the integrating particles, you understand that is the genome of the virus, once it is in contact with the nuclear material, changes the, nuclear, the real DNA particle of the cell, and once that kind of transcription occurs, you understand, then it affects the actual way that particular cell behaves, and that is actually the basis of turning the normal cells into a malignant cell but these things takes place over a long time, depending on the virulence, you understand, that is the attacking power of the virus, so the virulence of the organism and the dose of the organism, what I mean is that the amount of, the initial amount that the cell is in contact with and then the concentration, in terms of the volume that is affected, is actually what determines the rate of changes, and progression from the changes, you understand, so one cannot say this is, although in reality you can say probably 5 to 15 years, that’s the reason why, that’s the reason why most people that are targeted they are pre-pubertal girls because we expect that at least, they will not be exposed, it is their normal cell, it is their normal flora and even if they are in contact, that thing does not have the form of virulence attached to it, so, when you vaccinate at pre-pubertal or pubertal age, you expect the cover to enter into the reproductive age group, you understand, thereby preventing the development of the HPV infection

I: okay sir, do you know if there are strains, or something, like they say about the HIV virus, there are strains, that there are some strains that are more dangerous than the others

R:well, all I know, is that there could be some sub, there could be some sub types , you understand, there could be sub types of human papilloma virus, that one I don’t know, or I haven’t updated, or have not read it in the book in recent times, but definitely there are variants, variants, there are strains, and it is because there are strains that’s why some will have the HPV, they won’t develop the cancer, and some will have it they will develop, some they may even see everything but it is neither here nor there, you will find the virus there yet , every other thing will be normal, so what actually leads to that kind of a thing is both the host factor, as well as the agent factor

I: how do you mean sir?

R:what I mean is that the host factor is the human being that is trying to get the infection, whereas the agent factor is the agent itself, but you see, before any infection can be established, the host factor, host barriers has to be destroyed , the environment has to be favourable to the virus itself and then the virus has to multiply to the tune of at least 10^5^ before you can say there is an infection, there is a HPV infection, you understand, so that is just it

I: thank you very much sir, It has been really, really educating, sir what can you say about the HPV vaccine

R: ehm, what I can say about it is that ehm, the HPV vaccine, ehm, is better to be given by a trained person, you understand, it is better to be given by a trained person, , you know, we have in vaccination, there are some vaccination that could be active, there are some that could be passive, there are some that are attenuated, it depends on the, for now, I can’t say exactly which one that the HPV vaccine belongs to, probably until I see it and then I can tell you, and don’t forget that most of all these vaccines, it depends on the manufacturer, and then the manufacturer now depends, it depends on what the manufacturer actually wants to achieve, you understand, all I know, is that the HPV vaccine, is available to be taken, but one thing that is important is that , for any vaccination, number one, you have to maintain what we call the cold chain, secondly , it should be given by the trained expert, and thirdly there must be a good record, of all the vaccination that is taken, you understand, but one thing that is common to all, is that when a vaccine is taken, you want to watch for side effects, and then you want to watch for the therapeutic effects, the therapeutic effect is the prevention of the HPV, and another thing is that you must try as much as possible to maintain the potency of that vaccination and that is what I am talking about, that is the cold chain, the cold chain, the potency has to be kept, then you know that at least, the efficacy of the vaccine is maintained

I: sir, do you know anything about the types of vaccine available for now, the schedule of the vaccine

R: well, me for now, I have not taken too much concern about that, what I know is that in the immunization committee that we normally do, they normally order for the vaccine, now they leave it to the manufacturers, I think that the, the schedule, if you ask the public health nurses, they will be able to give you detailed, you understand, information on that area,

I: what do you think is the importance of that vaccine

R: the importance?

I: yes,

R: well, it is a good thing, it is important that one takes the HPV vaccine, because you see, in public health, you are not giving only one person, public health has to do with the population, you understand, and what you are looking at is the incidence of a particular illness in a population, and the kind of target group that you have to invite, that’s why you see cervical cancer is commoner in women of reproductive age, If cervical cancer affects only women of reproductive age ,then it requires targeting girls before they reach the reproductive age, you want to give it to them so that it will be prevented, what I mean is that the number of the incidence of that cervical cancer will be drastically reduced, it is more of a futuristic approach, those one that already have cervical cancer, there is nothing you can do for them, it has already happened, but now you can now say okay, let’s say 8% of the population or 6% of the population has cervical cancer, so you are targeting that in the next ten years, the cervical cancer, it is expected to be reduced to 2% or 1% , it may not be completely eradicated, so now you have known your target group, which are the target teenagers, young people, which are, or something like that so by the time you are vaccinating them now, you are looking at the result in 10 years time, you understand, your aim, is to ensure, to encourage as many number of people as possible, because if you don’t encourage as many people as possible, you understand, it will be that , those ones that didn’t go are the ones who will give issues, and then in 10 years time when you are supposed to get 1% , you still get 6%, the intervention has not worked, that’s just it

I: thank you very much for that sir, sir, do you know anything about recommendations for the vaccine in Nigeria,

R: recommendation for the vaccine?

I: yes

R: recommendation for the vaccine, I have not heard about that, [ like] no I know it’s highly recommended, you understand, for example from the gynaecology conferences and all those things you understand, gynaecologist, they have said at their conference workshops that it is highly recommended, for that is the pre pubertal girls, to have it, you understand, you understand, whether it is now a full policy by the federal government that all , everybody must have it is what I cannot say for now, I have not read about it

I: thank you very much sir, you know Nigeria has a program on immunization

R: yes

I: and then, if this vaccine is introduced into the routine immunization schedule, what do you think will be the benefit,

R: it is the benefit I have told you, there will be a drastic reduction

I: okay in the incidence of cervical cancer, asides that do you have any other thing to add

R: there will be increase in productivity , there will be increase,, those people that are sick, you understand, they are liability to the system, you understand, where as those people that are active, they are the productive people of the economy, you understand , when someone is, health is one of those intangible things that you see around, it is very difficult for you to measure it in naira and kobo, that’s why most of the things they do, you understand, but everybody knows that, you understand, overtime, if you are able to monitor your health, there is a significant increase in human development, usually human capacity development, you understand and once there is an increase in the human capacity development, it ultimately leads to increase in gross domestic product, and then the economy grows, and then there is a global increase in the quality of life of everybody, so that is it

I: thank you very much for that sir, what do you think will be the disadvantages, what will be the disadvantages of having the vaccine or any concerns

R: well disadvantages could also be, in form of cultural aspect, cultural aspect in part of the north, you understand, they are so sensitive about their girl child, and then, unless you explain everything to them fully, they may not , cooperate, you understand, and don’t forget that there is the sharia system they operate, they practice you understand, in this part of the country, the southwest, we don’t have ,ignorance, which may actually serve as a barrier, and also another thing that could serve as a barrier is cost implication, if the cost is too high and then people don’t see the benefit, sharp, sharp like this, now, you understand, you find out that when the patient, when they introduce the person who is going to take it, they will ask , of what benefit is this thing, you understand, so you have to start sitting down to let the parent know exactly the benefit they are going to derive from it, you understand, that is not something they can see by eyes but it something that as times goes on , everybody will appreciate it, you understand, so that’s just it, some area because of poverty too , you understand, I think that what Government can do in that realm to try as much as possible to put it under NHIS, so that at least out of pocket payment will not be there, and people will just take it

I: Sir, do you think that will work considering that everybody is not captured under NHIS

R:It’s possible that it works depending on the type of insurance policy the person has, if the person is, since the person is less than 18 years old, and is taken to that kind of thing, you know that what will happen is that, you know if you are in the formal sector now, there is a particular amount that they deduct from you as NHIS enrollee, and if you are in an informal sector, what they do now, is you create your cooperative system, and your cooperative system pays a certain amount for a year and with that, you should , another thing is that if it is captured by NHIS, the price, the out of pocket issue, will not be a disturbing issue for the person that wants to take it, you understand, other thing that can come up is that, the person that they are giving the vaccine must be well monitored, you have to watch that there is no allergy or something like that, so that is it

I: thank you sir, would you recommend this vaccine to an adolescent?

R: I have recommended, I do recommend it freely

I: can you share instances

R: well the , the , the, thing is that, adolescents generally, you understand, you run adolescent clinic, specifically for them, they won’t come to the clinic, to say that they want to take vaccine, you understand and this particular vaccine, is not popular among the people, what we do is that it is better to first of all take the advocacy to them , when you take the advocacy to them, and they are able to buy into the idea, they are the ones that will tell their other mates, their subordinates, that this thing is good, come and take it, then you now have a critical mass, for now, there is nothing like that, when they come, you understand, occasionally they come with other complaints, not that they come primarily for cervical vaccination, because there are so many things that disturbs an adolescent mind, peer group influence is there, his or her future is there, the source of finances is there, his hormones are there, that are always disturbing him or her, so also is way of life too, that’s why the first bet is take the advocacy to wherever they are, the youths , probably secondary school girls, arrange for, them, probably a talk, you understand for the, you tell them, when they know the importance, 30 to 50% of them can say okay, I want the vaccine, those who are saying yes, they are saying yes because they want to know more, you must be friendly with them, when you are friendly with them, they want to know, that there is no side effects on it, they will now be the person that will convince their mates to take it

I: okay sir, thank you very much sir, the advocacy you just mentioned, whose role do you think it is

R: the public health nurses

I: okay so they are the ones to go and tell people about it,

R: occasionally they can bring the doctor along, but that is their major work, to try to talk people into buying into health ideas

I: thank you very much sir, we have had an enlightening time here, this will be the end of the interview
