# Supplementary material for: Facilitators and barriers of healthcare workers’ recommendation of HPV vaccine for adolescents in Nigeria: views through the lens of theoretical domains framework
Source: BMC Health Serv Res. 2022 Jun 25;22:824. doi: 10.1186/s12913-022-08224-7 (PMC9233785; doi:10.1186/s12913-022-08224-7)
Supplement: Supplementary file 13 — Additional file 13. [file 12913_2022_8224_MOESM13_ESM.docx]

**INTERVIEW ID:**

**TYPE OF INTERVIEW: IDI**

**PARTICIPANT: CONSULTANT PAEDIATRICIAN, UNIVERSITY COLLEGE HOSPITAL, IBADAN**

**NUMBER OF PARTICIPANT: 1**

**INTERVIEWER: T**

**TIME OF INTERVIEW: 14:20**

**LANGUAGE OF INTERVIEW: ENGLISH**

**VENUE OF INTERVIEW: UCH, IBADAN**

**AGE OF PARTICPANT: 59**

**GENDER: FEMALE**

**DATE OF INTERVIEW:**

I: good afternoon ma, my name is XXXXXXX Owolabi, I am here to ask you some questions about your knowledge of cervical cancer, Human papilloma virus and Human papilloma virus vaccine, and I want to assure you that everything you will be saying to us is confidential, we don’t need your name but we just need some other information about you, basically your socio demographics, thank you very much ma, I will like you to tell me about yourself, like how old are you ma

R: I am 59

I: 59, like how long have you been working, what post, what is your designation

R: I am consultant paediatrician,

I: how long have you been working

R: that’s 16 years

I: 16 years, thank you very much ma, I will like to know, what do you know about cervical cancer

R: what I know about cervical cancer, cervical cancer starting with the burden, it’s world wide but know that there is a predilection for black people, [okay] predilection for, of course it is only women that have cervix, it is a female, exclusively female cancer, and it is being linked one of the things that has been associated with it, is people that have had sexual experience, in other words, people who have not had sexual experience are unlikely to have cervical cancer, and it has also been linked with a virus, human papilloma virus, so the virus, has serotypes,, some common ones have been linked with cervical cancer also some linked with anal cancer that can affect both men and women but cervical cancer actually, there are particular serotypes that are commoner and that’s why the HPV vaccine was invented against, especially those serotypes, that’s why they were invented against those serotypes, so the HPV Vaccine is a world celebrated vaccine because cervical cancer is the one cancer that has prevention that’s why the HPV vaccine is welcomed and is well celebrated, and It is been introduced in the UK, in Europe and America and funny enough, some parts in Africa, Rwanda for instance, South Africa introduced it in 2014 or so, as routine immunization, so we are lagging, it can be sourced here privately but you know private vaccine is expensive , but the Nigeria program, the immunization program has it in mind, it is in the pipeline, they will introduce it after they have introduced the more urgent, more burden vaccines, it’s in the pipeline, that’s what we have been told

I: so it is not a high burden vaccine

R: it is, you know if you are talking about a vaccine, the age of the affected, the number of people affected, the cost of this vaccine, and the WHO rightly give this guideline and each country is to decide, some of those cost, they decide which vaccine they should introduce one by one they decide, which one to introduce first and which one to take next, they have accepted that it is a very useful vaccine and has put it in the pipeline, it is available even in UCH here, it is available, it was initially three doses, 0 ,6 months and 1 year. Studies have shown, and the age group, from 9, 9 to 10 years, now many countries have decided to widen theirs, between 9 ad 14, it also captures those who have not had any sexual debut, studies have shown that, you don’t even need three doses, just 2 now, so, it has been rescheduled to be just 2 doses and, that’s all

I: apart from the vaccine, are there other ways by which we can prevent cervical cancer

R: prevent cervical cancer early detection by pap smear, that’s what has been introduced in medical practice for women over 35, I mean, because a cancer does not just advance overnight. It has a style, 1,2,3, advance like that, and the pap smear program is done well and women really take to it, when its detected at the early, pre cancer stage, one can nip it in the bud, but of course when it is already advanced, it is difficult to nip it in the bud, but the best prevention is immunization

I: ma, have you been exposed to any training on cervical cancer

R:hmm, I have had a lot

I: okay, at what point,

R: emm, after I became a consultant, I did a vaccinology course

I: but apart from that you have not had any training

R: that was just 2 years ago

I: 2 years ago, so it is still fresh [ laughter}, alright ma, so what do you think will be the benefit, if this vaccine is introduced into the routine schedule of immunization,

R: for Nigeria, that’s what we said, if we have this cancer that has a high burden among, right now, it should actually be number 1, well its struggling with breast cancer, sometimes, one will overtake the other but it is a very high burden problem, cancer of the cervix, and because it has a solution, a prevention, like a preventive measure, that’s why one should recommend the immunization, the vaccine

I:,okay, do you think that there will be any disadvantages if it is introduced

R: it is when people have not been informed appropriately, you have not given enough awareness, it is all the rumours and all the misconceptions about the vaccines people have said, it is a license to become promiscuous when you educate people about the benefit of the vaccine, about the cancer itself, people start to benefit by being immunised against the cancer, then I think it should be accepted then

I: ma, the issue you just mentioned now that it make people to become more promiscuous, is that something that is playing presently,

R: when I did the vaccinology course, I got to know when we did our scenario and all these type of thing, it was not in Nigeria, people came to make presentations of their thesis,, when they did pilot, because you have to do pilot in some of these countries and they did the pre- introduction assessment, those were the things that came out

I: ma do you think there may be any challenge if this vaccine is introduced may be manpower, or relating to the vaccine itself, do you envisage any challenge,

R:it is the cost, because if it is going to be, that’s why I was saying some other vaccine, it’s been argued that it needs to be introduced first, but when it reaches the turn of HPV, I mean funds are there and it’s produced, we must make sure we educate the community enough about it, so that we won’t have any rejections that is related to that

I: so once we do that

R: I think we are fine, I think it’s okay, we won’t have any problem, as for side effect, I can’t say if there was much at all, any side effect, but recently I was reading something about, something came up recently, something came up in the news about HPV, but of course more studies are coming up because of that initial , I can’t remember anymore, but there are some worries recently about HPV, so there are studies trying to access that, to really know if there are any associations or not, you know of course that when a vaccine is introduced, you have to do a post licencing , you have to do based on, you have to keep looking for side effects and issues when this vaccine has been introduced, I think that was what brought about what we have been saying, I think, I can’t just remember now, to prove or disprove, any length, any association with the vaccine

I: ma, have you had to recommend this vaccine to people

R: not personally, but I belong to the immunization committee in the hospital and its one of the vaccine that is stocked, as non-routine, its stocked, so indirectly you say , I have recommended, it’s a good thing and so the committee recommended, you write a letter to say ,this vaccine is available, please avail the eligible, and children of this vaccine

I: is there any reason why you will not recommend this vaccine,

R:I don’t think so , I don’t think so, I am trying to remember any major side effect, I don’t think there should be any major effect, so these are things that should

I:, so you don’t have any reservation, do you have any recommendation, or anything, that may be of help to the study

R: I need to remember this issue that has been linked with HPV, its going on, in fact its making the rounds, in the last one month or so, it’s been going round, so I will look it up again , see what that matter is, and that’s what I will now recommend and say, since It is an added issue from all the misconception and rumours, better to deal with those issues to see if they are really linked with HPV before countries that are , otherwise, it Is recipe for disaster for us, for the other routine vaccines, we are not doing too well in the coverage, if we have this additional issues, then we won’t have good coverage, we won’t have good acceptance

I:thank you very much for your time ma, that will be all
