# Supplementary material for: Facilitators and barriers of healthcare workers’ recommendation of HPV vaccine for adolescents in Nigeria: views through the lens of theoretical domains framework
Source: BMC Health Serv Res. 2022 Jun 25;22:824. doi: 10.1186/s12913-022-08224-7 (PMC9233785; doi:10.1186/s12913-022-08224-7)
Supplement: Supplementary file 15 — Additional file 15. [file 12913_2022_8224_MOESM15_ESM.docx]

SUPPLEMENT

1: COREQ 32--‐ITEM CHECKLIST Tong A Sainsbury P, Craig J. (2007) Consolidated criteria for reporting qualitative research (COREQ): a 32--‐ item checklist for interviews and focus groups. International Journal for Quality in Healthcare: 19:349 – 357.

| S/N | Item | Guide questions/description | Reported on Page No |
| --- | --- | --- | --- |
|  | Domain 1: Research team and reflexivity |  |  |
| 1. | Interviewer/facilitator | Which author/s conducted the interview? | 8 |
| 2. | Credentials | What were the researcher’s credentials? | 8 |
| 3. | Occupation | What was their occupation at the time of the study? | 8 |
| 4. | Gender | Was the researcher male or female? | 8 |
| 5. | Experience and training | What experience or training did the researcher  have? | 8 |
| 6. | Relationship with participants established | Was a relationship established prior to study  commencement? | 8 |
| 7. | Participant knowledge  Of the interviewer | What did the participants know about the  researcher? | 8 |
| 8. | Interviewers’ characteristics | What characteristics were reported about the  interviewer/facilitator? | 8 |
|  | Domain 2: Study design |  |  |
| 9. | Methodological orientation and Theory | What methodological orientation was stated to underpin the study? | 5-6 |
| 10. | Sampling | How were participants selected? | 7 |
| 11. | Method of approach | How were participants approached? | 7-8 |
| 12. | Sample size | How many participants were in the study? | 7-8 |
| 13. | Non participation | How many people refused to participate or dropped out? Reasons? | 8 |
| 14. | Setting of data collection | Where was the data collected? | 8 |
| 15. | Presence of non-participants | Was anyone else present besides the participants and researchers? | 8 |
| 16. | Description of sample | What are the important characteristics of the  sample? | 10 |
| 17. | Interview guide | Were questions, prompts, guides provided by the authors? | 8 |
| 18. | Repeat interviews | Were repeat interviews carried out? | 8 |
| 19. | Audio/visual recording | Did the research use audio or visual recording to collect the data? | 8 |
| 20. | Field notes | Were field notes made during and/or after the interview? | 8 |
| 21. | What was the duration of the interviews? | What was the duration of the interviews | 8 |
| 22. | Data saturation | Was data saturation discussed? | 8 |
| 23. | Transcripts returned | Were transcripts returned to participants for comment and/or correction? | No |
| 24. | Number of data coders | How many data coders coded the data? | 8 |
| 25. | Description of the coding tree | Did authors provide a description of the coding  tree? | 8 |
| 26. | Derivation of themes | Were themes identified in advance or derived from the data? | 9 |
| 27. | Software | What software, if applicable, was used to manage the data? | None |
| 28. | Participant checking | Did participants provide feedback on the  findings? | No |
| 29. | Quotations presented | Were participant quotations presented to illustrate the themes/findings? Was each quotation identified? | 11, 14, 23 |
| 30. | Data and findings consistent | Was there consistency between the data presented and the findings? | 10-15, 23 |
| 31. | Clarity of major themes | Were major themes clearly presented in the  findings? | 10-15, 23 |
| 32. | Clarity of minor themes | Is there a description of diverse cases or  Discussion of minor themes? | 10-15, 23 |
